# Sex-Specific Thresholds of High-Sensitivity Troponin in Patients With Suspected Acute Coronary Syndrome

**DOI:** 10.1016/j.jacc.2019.07.082

**Published:** 2019-10-22

**Authors:** Kuan Ken Lee, Amy V. Ferry, Atul Anand, Fiona E. Strachan, Andrew R. Chapman, Dorien M. Kimenai, Steven J.R. Meex, Colin Berry, Iain Findlay, Alan Reid, Anne Cruickshank, Alasdair Gray, Paul O. Collinson, Fred S. Apple, David A. McAllister, Donogh Maguire, Keith A.A. Fox, David E. Newby, Chris Tuck, Catriona Keerie, Christopher J. Weir, Anoop S.V. Shah, Nicholas L. Mills, High-STEACS Investigators, Nicholas L. Mills, Fiona E. Strachan, Christopher Tuck, Anoop S.V. Shah, Atul Anand, Amy V. Ferry, Kuan Ken Lee, Andrew R. Chapman, Dennis Sandeman, Philip D. Adamson, Catherine L. Stables, Catalina A. Vallejo, Athanasios Tsanasis, Lucy Marshall, Stacey D. Stewart, Takeshi Fujisawa, Mischa Hautvast, Jean McPherson, Lynn McKinlay, Nicholas L. Mills, David E. Newby, Keith A.A. Fox, Colin Berry, Simon Walker, Christopher J. Weir, Ian Ford, Nicholas L. Mills, David E. Newby, Alasdair Gray, Keith A.A. Fox, Colin Berry, Simon Walker, Paul O. Collinson, Fred S. Apple, Alan Reid, Anne Cruikshank, Iain Findlay, Shannon Amoils, David A. McAllister, Donogh Maguire, Jennifer Stevens, John Norrie, Christopher Weir, Anoop S.V. Shah, Atul Anand, Andrew R. Chapman, Kuan Ken Lee, Jack P.M. Andrews, Philip D. Adamson, Alastair Moss, Mohamed S. Anwar, John Hung, Nicholas L. Mills, Simon Walker, Jonathan Malo, Alan Reid, Anne Cruikshank, Paul O. Collinson, Colin M. Fischbacher, Bernard L. Croal, Stephen J. Leslie, Catriona Keerie, Richard A. Parker, Allan Walker, Ronnie Harkess, Christopher Tuck, Tony Wackett, Christopher Weir, Roma Armstrong, Marion Flood, Laura Stirling, Claire MacDonald, Imran Sadat, Frank Finlay, Heather Charles, Pamela Linksted, Stephen Young, Bill Alexander, Chris Duncan

**Affiliations:** a British Heart Foundation Centre for Cardiovascular Science, University of Edinburgh, Edinburgh, United Kingdom; b CARIM School for Cardiovascular Diseases, Maastricht University, Maastricht, the Netherlands; c Central Diagnostic Laboratory, Maastricht University Medical Center, Maastricht, the Netherlands; d Institute of Cardiovascular and Medical Sciences, University of Glasgow, Glasgow, United Kingdom; e Department of Cardiology, Royal Alexandra Hospital, Paisley, United Kingdom; f Department of Biochemistry, Queen Elizabeth University Hospital, Glasgow, United Kingdom; g Emergency Medicine Research Group Edinburgh, Royal Infirmary of Edinburgh, Edinburgh, United Kingdom; h Departments of Clinical Blood Sciences and Cardiology, St. George’s, University Hospitals NHS Trust and St. George’s University of London, London, United Kingdom; i Department of Laboratory Medicine and Pathology, Hennepin County Medical Center & University of Minnesota, Minneapolis, Minnesota; j Institute of Health and Wellbeing, University of Glasgow, Glasgow, United Kingdom; k Emergency Medicine Department, Glasgow Royal Infirmary, Glasgow, United Kingdom; l Edinburgh Clinical Trials Unit, University of Edinburgh, Edinburgh, United Kingdom; m Usher Institute of Population Health Sciences and Informatics, University of Edinburgh, Edinburgh, United Kingdom

**Keywords:** acute coronary syndrome, high-sensitivity cardiac troponin, myocardial infarction, sex-specific threshold, CI, confidence interval, cTnI, contemporary cardiac troponin I, HR, hazard ratio, hs-cTnI, high-sensitivity cardiac troponin I

## Abstract

**Background:**

Major disparities between women and men in the diagnosis, management, and outcomes of acute coronary syndrome are well recognized.

**Objectives:**

The aim of this study was to evaluate the impact of implementing a high-sensitivity cardiac troponin I assay with sex-specific diagnostic thresholds for myocardial infarction in women and men with suspected acute coronary syndrome.

**Methods:**

Consecutive patients with suspected acute coronary syndrome were enrolled in a stepped-wedge, cluster-randomized controlled trial across 10 hospitals. Myocardial injury was defined as high-sensitivity cardiac troponin I concentration >99th centile of 16 ng/l in women and 34 ng/l in men. The primary outcome was recurrent myocardial infarction or cardiovascular death at 1 year.

**Results:**

A total of 48,282 patients (47% women) were included. Use of the high-sensitivity cardiac troponin I assay with sex-specific thresholds increased myocardial injury in women by 42% and in men by 6%. Following implementation, women with myocardial injury remained less likely than men to undergo coronary revascularization (15% vs. 34%) and to receive dual antiplatelet (26% vs. 43%), statin (16% vs. 26%), or other preventive therapies (p < 0.001 for all). The primary outcome occurred in 18% (369 of 2,072) and 17% (488 of 2,919) of women with myocardial injury before and after implementation, respectively (adjusted hazard ratio: 1.11; 95% confidence interval: 0.92 to 1.33), compared with 18% (370 of 2,044) and 15% (513 of 3,325) of men (adjusted hazard ratio: 0.85; 95% confidence interval: 0.71 to 1.01).

**Conclusions:**

Use of sex-specific thresholds identified 5 times more additional women than men with myocardial injury. Despite this increase, women received approximately one-half the number of treatments for coronary artery disease as men, and outcomes were not improved. (High-Sensitivity Troponin in the Evaluation of Patients With Acute Coronary Syndrome [High-STEACS]; NCT01852123).

Important disparities exist in the diagnosis, management, and outcomes of acute coronary syndrome between women and men 1, 2, 3. Women with suspected acute coronary syndrome are less likely to undergo evidence-based investigations and treatment, and outcomes following myocardial infarction are consistently poorer compared with men (1).

The development of high-sensitivity cardiac troponin assays has resulted in the identification of important sex differences in the reference range of cardiac troponin, with the 99th centile in men being twice that in women 4, 5, 6. We have previously demonstrated that the use of a high-sensitivity assay with sex-specific thresholds may double the diagnosis of myocardial infarction in women and identify women at risk for future cardiac events (7). This raises the question as to whether the use of single diagnostic thresholds has contributed to inequalities in the diagnosis, management, and outcomes of women with acute coronary syndrome.

The recently published fourth universal definition of myocardial infarction recommends the use of sex-specific thresholds for the diagnosis of myocardial infarction (2). High-STEACS (High-Sensitivity Troponin in the Evaluation of Patients With Suspected Acute Coronary Syndrome) was the first randomized controlled trial to evaluate the introduction of a high-sensitivity cardiac troponin I (hs-cTnI) assay with sex-specific thresholds into clinical practice (8). The hs-cTnI assay reclassified 1 in 6 patients with myocardial injury, but this was not associated with a reduction in recurrent myocardial infarction or cardiovascular death at 1 year. In this pre-specified secondary analysis, we evaluated the impact of implementing sex-specific diagnostic thresholds on the use of investigation and treatments for coronary heart disease and on clinical outcomes in women and men separately.

## Methods

### Study design

High-STEACS was a stepped-wedge, cluster-randomized controlled trial that evaluated the implementation of an hs-cTnI assay in consecutive patients presenting with suspected acute coronary syndrome across 10 secondary and tertiary hospitals in Scotland (NCT01852123) (Online Table 1). The study design has been described in detail previously (8), and the study was conducted with the approval of the Scotland Research Ethics Committee in accordance with the Declaration of Helsinki. During a validation phase of at least 6 months, a contemporary cardiac troponin I (cTnI) assay was used to guide clinical care. Hospital sites were then randomly allocated to early or late implementation of the hs-cTnI assay and sex-specific thresholds. Hospital sites served as the unit of randomization to avoid the risk for clinical error due to reporting of different troponin assays and thresholds simultaneously.

### Patient population

All patients presenting to the emergency department were screened by the attending clinician for suspected acute coronary syndrome using an electronic form embedded within the clinical care pathway. Patients who presented with suspected acute coronary syndrome and had paired troponin measurements using the contemporary and trial assay were eligible for inclusion. Patients who were not resident in Scotland or who had previously been admitted during the trial period were excluded. Because the intervention was implemented at the hospital level, individual patient consent was not sought. This approach ensured that consecutive patients presenting with suspected acute coronary syndrome were included without selection bias.

### Intervention

Cardiac troponin testing was performed at presentation and repeated 6 or 12 h after the onset of symptoms at the discretion of the attending clinician in accordance with national and international guidelines in use during enrollment 9, 10. Throughout the duration of the trial, all sites measured cardiac troponin using both the cTnI and hs-cTnI assays simultaneously. During the validation phase, only the results of the cTnI assay were reported to the attending clinician, while during the implementation phase, only the results of the hs-cTnI assay were reported.

The cTnI assay (ARCHITECT STAT troponin I assay; Abbott Laboratories, Abbott Park, Illinois) with a single diagnostic threshold for women and men was used to guide clinical decisions during the validation phase. The interassay coefficient of variation was <10% at 40 ng/l at 7 sites and 50 ng/l at 3 sites, and these concentrations were used as the diagnostic thresholds during the validation phase (11). During the implementation phase, an hs-cTnI assay (ARCHITECT STAT high-sensitive troponin I assay; Abbott Laboratories) was used to guide clinical decisions, with a sex-specific 99th centile diagnostic threshold of 16 ng/l for women and 34 ng/l for men (7).

### Adjudication of the diagnosis of myocardial infarction

Two physicians from our adjudication panel independently reviewed all clinical information in those with hs-cTnI concentrations >99th centile while blinded to the study phase to classify patients in accordance with the third universal definition of myocardial infarction (12). Any disagreements were resolved by a third physician (Online Appendix).

### Outcomes

We used regional and national registries to ensure complete follow-up of the trial population 7, 13, 14. The primary outcome was a composite of type 1 or type 4b myocardial infarction following the initial presentation to hospital or cardiovascular death within 1 year. The primary outcome was independently adjudicated by 2 physicians blinded to study phase, and any disagreements were resolved by a third physician.

### Statistical analysis

All patients with peak hs-cTnI above the sex-specific 99th centile were classified as having myocardial injury. Patients with myocardial injury were further stratified into 2 groups: those already identified by the contemporary cTnI assay (cTnI concentrations above the diagnostic threshold of the contemporary assay) and those reclassified by the hs-cTnI assay (cTnI concentrations below the diagnostic threshold of the contemporary assay). The primary outcome was compared before and after implementation of the hs-cTnI assay stratified by sex in those with myocardial injury using a Cox proportional hazards model. The model adjusted for hospital site (fitted as a random effect), season, time of presentation from the start date of the trial, age, sex and study phase as an interaction term, history of diabetes mellitus, ischemic heart disease or cerebrovascular disease, hs-cTnI, creatinine concentration, and social deprivation. We used the same model to compare the primary outcome in women and men reclassified by the hs-cTnI assay and in post hoc analyses (Online Appendix). We performed a post hoc analysis in women, stratified by the uniform 99th centile diagnostic threshold (26 ng/l). All statistical analyses were performed using R version 3.5.1 (R Foundation, Vienna, Austria).

## Results

Consecutive patients (n = 48,282) with suspected acute coronary syndrome were included in this trial, of whom 22,562 (47%) were women and 25,720 (53%) were men (Online Figure 1). The majority of patients reclassified by the hs-cTnI assay and sex-specific thresholds were women (1,470 of 1,771 women [83%] vs. 301 of 1,771 men [17%]), with the total number of patients with myocardial injury increasing from 3,521 (16%) to 4,991 (22%) in 22,562 women and from 5,068 (20%) to 5,369 (21%) in 25,720 men.

### Baseline characteristics

Across both study phases, women with myocardial injury were older than men (75 ± 14 years vs. 68 ± 15 years), although they had similar cardiovascular risk factors, pre-existing medications, and risk for mortality (GRACE [Global Registry of Acute Coronary Events] score 147 ± 36 vs. 140 ± 39) (Table 1). Women were as likely as men to present with chest pain (66% vs. 74%) and to have evidence of myocardial ischemia on electrocardiography (27% vs. 36%). Compared with those identified by the cTnI assay, both women and men reclassified by the hs-cTnI assay were less likely to have myocardial ischemia on electrocardiography but had similar age, presenting symptoms, and cardiovascular risk factors (Online Table 2).

**Table 1:** Characteristics of Trial Participants With Myocardial Injury, Stratified by Sex and Study Phase

	Overall (N = 10,360)	Women			Men		
		Overall (N = 4,991)	Validation (n = 2,072)	Implementation (n = 2,919)	Overall (N = 5,369)	Validation (n = 2,044)	Implementation (n = 3,325)
Age, yrs	71 ± 15	75 ± 14	76 ± 14	74 ± 14	68 ± 15	68 ± 15	67 ± 15
Presenting symptom∗							
Chest pain	6,449 (70)	2,880 (66)	940 (64)	1,940 (67)	3,569 (74)	1,076 (72)	2,493 (75)
Dyspnea	1,068 (12)	575 (13)	192 (13)	383 (13)	493 (10)	173 (12)	320 (10)
Palpitation	278 (3)	172 (4)	58 (4)	114 (4)	106 (2)	37 (3)	69 (2)
Syncope	686 (7)	388 (9)	153 (10)	235 (8)	298 (6)	109 (7)	189 (6)
Other	730 (8)	374 (9)	128 (9)	246 (8)	356 (7)	102 (7)	254 (8)
Previous medical conditions							
Myocardial infarction	1,379 (13)	631 (13)	291 (14)	340 (12)	748 (14)	325 (16)	423 (13)
Ischemic heart disease	3,457 (33)	1,663 (33)	773 (37)	890 (31)	1,794 (33)	758 (37)	1,036 (31)
Cerebrovascular disease	1,034 (10)	573 (12)	265 (13)	308 (11)	461 (9)	190 (9)	271 (8)
Diabetes mellitus	1,478 (14)	664 (13)	282 (14)	382 (13)	814 (15)	329 (16)	485 (15)
Previous revascularization							
PCI	938 (9)	355 (7)	148 (7)	207 (7)	583 (11)	229 (11)	354 (11)
CABG	248 (2)	76 (2)	35 (2)	41 (1)	172 (3)	76 (4)	96 (3)
Medications at presentation							
Aspirin	3,701 (36)	1,759 (35)	800 (39)	959 (33)	1,942 (36)	809 (40)	1,133 (34)
P2Y_12_ inhibitor	1,422 (14)	750 (15)	357 (17)	393 (14)	672 (13)	296 (15)	376 (11)
Dual-antiplatelet therapy†	502 (5)	228 (5)	121 (6)	107 (4)	274 (5)	139 (7)	135 (4)
Statin	5,260 (51)	2,499 (50)	1,100 (53)	1,399 (48)	2,761 (51)	1,097 (54)	1,664 (50)
ACE inhibitor or ARB	4,333 (42)	2,059 (41)	876 (42)	1,183 (41)	2,274 (42)	892 (44)	1,382 (42)
Beta-blocker	3,607 (35)	1,809 (36)	794 (38)	1,015 (35)	1,798 (34)	762 (37)	1,036 (31)
Oral anticoagulant agent‡	1,095 (11)	587 (12)	269 (13)	318 (11)	508 (10)	204 (10)	304 (9)
Loop diuretic agent	2,693 (26)	1,571 (32)	702 (34)	869 (30)	1,122 (21)	491 (24)	631 (19)
Proton-pump inhibitor	4,638 (45)	2,472 (50)	1,084 (52)	1,388 (48)	2,166 (40)	862 (42)	1,304 (39)
Calcium-channel blocker	1,977 (19)	921 (19)	397 (19)	524 (18)	1,056 (19)	412 (20)	644 (19)
Nicorandil	645 (6)	303 (6)	149 (7)	154 (5)	342 (6)	174 (8)	168 (5)
Ivabradine	146 (1)	68 (1)	25 (1)	43 (1)	78 (1)	33 (1)	45 (1)
Spironolactone	450 (4)	201 (4)	82 (4)	119 (4)	249 (4)	113 (5)	136 (4)
Electrocardiographic results§							
Normal	2,672 (34)	1,366 (36)	513 (36)	853 (36)	1,306 (32)	479 (34)	827 (30)
Myocardial ischemia	2,510 (32)	1,023 (27)	342 (24)	681 (28)	1,487 (36)	445 (32)	1,042 (38)
ST-segment elevation	998 (13)	329 (9)	90 (6)	239 (10)	669 (16)	174 (12)	495 (18)
ST-segment depression	1,328 (17)	583 (16)	226 (16)	357 (15)	745 (18)	234 (17)	511 (18)
T-wave inversion	1,277 (16)	640 (17)	252 (17)	388 (16)	637 (15)	232 (16)	405 (15)
Physiological parameters§							
Heart rate, beats/min	86 ± 26	88 ± 27	88 ± 27	88 ± 26	84 ± 26	85 ± 25	83 ± 26
Systolic blood pressure, mm Hg	139 ± 29	141 ± 30	140 ± 29	141 ± 30	137 ± 28	136 ± 28	137 ± 28
GRACE risk score	143 ± 38	147 ± 36	148 ± 34	147 ± 38	140 ± 39	139 ± 38	140 ± 40
Hematologic and clinical chemistry measurements							
Hemoglobin, g/l	131 ± 25	125 ± 24	124 ± 24	126 ± 23	137 ± 25	136 ± 25	137 ± 25
eGFR, ml/min	47 ± 16	46 ± 16	47 ± 16	46 ± 16	49 ± 15	49 ± 16	48 ± 15
Peak hs-cTnI, ng/l	158 (45–1,622)	82 (30–656)	69 (28–464)	93 (32–831)	294 (67–3,006)	216 (57–1,706)	404 (76–4,395)
Serial hs-cTnI, %‖	6,983 ± 67	3,230 ± 65	1,298 ± 63	1,932 ± 66	3,753 ± 70	1,411 ± 69	2,342 ± 70
Adjudicated diagnosis¶							
Type 1 MI	5,028 (49)	2,010 (46)	748 (44)	1,262 (47)	3,018 (64)	1,059 (62)	1,959 (65)
Type 2 MI	1,260 (12)	700 (16)	275 (16)	425 (16)	560 (12)	187 (11)	373 (12)
Nonischemic myocardial injury	2,810 (27)	1,673 (38)	670 (40)	1,003 (37)	1,137 (24)	448 (22)	689 (21)

Values are mean ± SD, n (%), or median (interquartile range).

ACE = angiotensin-converting enzyme; ARB = angiotensin receptor blocker; CABG = coronary artery bypass grafting; eGFR = estimated glomerular filtration rate; GRACE = Global Registry of Acute Coronary Events; hs-cTnI = high sensitivity cardiac troponin I; MI = myocardial infarction; PCI = percutaneous coronary intervention.

∗ Presenting symptom was missing in 5,615 patients (12%).

† Two medications from aspirin, clopidogrel, prasugrel, or ticagrelor.

‡ Includes warfarin and direct oral anticoagulant agents.

§ Electrocardiographic and physiological data were available in 1,377 of reclassified patients (78%) and 6,470 of identified patients (75%).

‖ Defined as 2 or more tests within 24 h of presentation.

¶ The adjudication panel was able to achieve consensus diagnoses in 9,098 of 10,360 patients (88%) with hs-cTnI concentrations above the sex-specific 99th centile.

### Diagnosis of myocardial infarction during index admission

**Figure 1:**
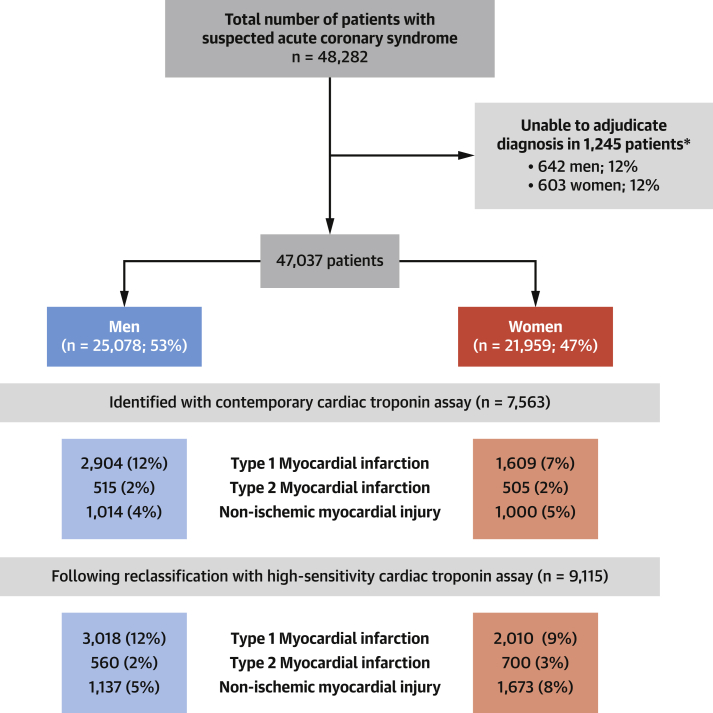
Flow Diagram of Adjudication Process in Women and Men With Suspected Acute Coronary Syndrome Adjudicated diagnoses are presented for patients with troponin concentration above the contemporary cardiac troponin I assay threshold of 50 ng/l and those with troponin concentration above the sex-specific 99th centile threshold of 16 ng/l in women and 34 ng/l in men. *Where there was consensus among the adjudication panel that there was insufficient clinical information to make a definitive diagnosis, because of missing admission or discharge letters, we did not attempt to adjudicate the diagnosis (1,245 of 10,360 [12%]). As we had access to all other information, including medical history, clinical investigations, management, and outcomes, these patients were not excluded from our primary or secondary analyses.

The adjudication panel was able to achieve a consensus diagnosis in 9,115 of 10,360 patients (88%) with hs-cTnI concentrations above the sex-specific 99th centile (Figure 1). The proportions of women and men with myocardial injury due to type 1 myocardial infarction were 52% (1,609 of 3,118) and 65% (2,904 of 4,445) in those identified by the cTnI assay and 32% (401 of 1,270) and 40% (114 of 282) in those reclassified by the hs-cTnI assay (Online Table 2). Overall, the use of an hs-cTnI assay and sex-specific thresholds increased the diagnosis of type 1 myocardial infarction in women by 25% (from 1,609 of 21,959 [7%] to 2,010 of 21,959 [9%]) and in men by 6% (from 2,904 of 25,078 [12%] to 3,018 of 25,078 [12%]) (Figure 1). The use of an hs-cTnI assay and sex-specific thresholds increased the diagnosis of type 2 myocardial infarction in women by 39% (from 505 of 21,959 [2%] to 700 of 21,959 [3%]) and in men by 9% (from 515 of 25,078 [2%] to 560 of 25,078 [2%]) and increased the diagnosis of nonischemic myocardial injury in women by 67% (from 1,000 of 21,959 [5%] to 1,673 of 21,959 [8%]) and in men by 12% (from 1,014 of 25,078 [2%] to 1,137 of 25,078 [2%]).

### Management of women and men during index admission

Women with myocardial injury presenting in the implementation phase were more likely than those presenting during the validation phase to undergo coronary angiography (18% vs. 26%) and coronary revascularization (10% vs. 15%) (p < 0.001 for both) (Table 2, Online Tables 3 and 4). Similarly, in men with myocardial injury, coronary angiography and revascularization increased from the validation to implementation phase (38% vs. 46% and 26% vs. 34%, respectively; p < 0.001 for both). However, across both phases, rates of coronary angiography and revascularization were lower in women compared with men (p < 0.001 for both). Likewise, prescriptions for preventive therapies increased following implementation in both women and men but across both phases remained lower in women (Table 2). These differences in management between women and men were also observed in patients older and younger than the median age of 73 years (Online Figure 2).

**Table 2:** Management of Patients With Myocardial Injury During Initial Hospital Admission, Stratified by Sex and Study Phase

	Overall (N = 10,360)	Women				Men			
		Overall (N = 4,991)	Validation (n = 2,072)	Implementation (n = 2,919)	p Value∗	Overall (N = 5,369)	Validation (n = 2,044)	Implementation (n = 3,325)	p Value∗
Duration of stay, h	74 (23–165)	76 (21–186)	71 (8–195)	79 (28–177)	<0.001	73 (26–146)†	75 (14–153)	72 (31–142)	0.247
Coronary angiography	3,425 (33)	1,120 (22)	367 (18)	753 (26)	<0.001	2,305 (43)†	770 (38)	1,535 (46)	<0.001
PCI	2,162 (21)	624 (13)	194 (9)	430 (15)	<0.001	1,538 (29)†	472 (23)	1,066 (32)	<0.001
CABG	158 (2)	31 (1)	11 (1)	20 (1)	0.617	127 (2)†	54 (3)	73 (2)	0.341
PCI or CABG	2,315 (22)	654 (13)	205 (10)	449 (15)	<0.001	1,661 (31)†	524 (26)	1,137 (34)	<0.001
New antiplatelet drug	4,094 (40)	1,580 (32)	595 (29)	985 (34)	<0.001	2,514 (47)†	877 (43)	1,637 (49)	<0.001
New DAPT	3,383 (33)	1,225 (25)	453 (22)	772 (26)	<0.001	2,158 (40)†	726 (36)	1,432 (43)	<0.001
New statin therapy	2,034 (20)	704 (14)	237 (11)	467 (16)	<0.001	1,330 (25)†	455 (22)	875 (26)	0.001
New ACE inhibitor or ARB	1,945 (19)	685 (14)	255 (12)	430 (15)	0.016	1,260 (24)†	450 (22)	810 (24)	0.055
New beta-blocker	2,559 (25)	960 (19)	322 (16)	638 (22)	<0.001	1,599 (30)†	571 (28)	1,028 (31)	0.023
New oral anticoagulant agent	643 (6)	313 (6)	119 (6)	194 (7)	0.216	330 (6)	117 (6)	213 (6)	0.346

Values are median (interquartile range) or n (%).

DAPT = dual-antiplatelet therapy; other abbreviations as in Table 1.

∗ Chi-square and Mann-Whitney *U*-tests comparing the validation and implementation phases.

† p < 0.05 comparing women and men.

Across both phases, women with type 1 myocardial infarction were less likely than men to undergo coronary angiography (43% vs. 66% during the validation phase and 53% vs. 73% during the implementation phase; p < 0.001 for both) and coronary revascularization (26% vs. 48% and 35% vs. 57%, respectively; p < 0.001 for both) (Online Table 5, Online Figure 3). They were also less likely than men to receive prescriptions for secondary prevention such as dual-antiplatelet therapy (48% vs. 61% during the validation phase and 54% vs. 67% during the implementation phase; p < 0.001 for both), statins (24% vs. 37% and 31% vs. 41%, respectively; p < 0.001 for both), and beta-blockers (26% vs. 42% and 33% vs. 42%, respectively; p < 0.001 for both).

**Figure 2:**
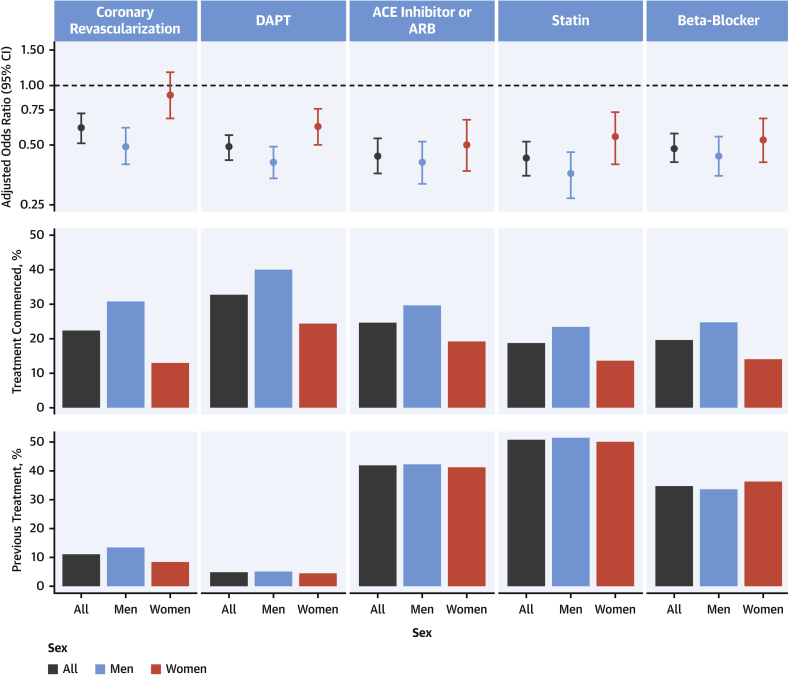
Patient Management During Index Hospitalization and Adjusted Odds Ratio of Myocardial Infarction or Cardiovascular Death at 1 Year Stratified by Treatment Received During Index Hospitalization and Sex **(Top)** Odds ratio of myocardial infarction and cardiovascular death at 1 year in patients receiving each treatment modality compared with those not receiving treatment for all patients and stratified by sex. Odds ratios were adjusted for hospital site (fitted as a random effect), season, time of presentation from the start date of the trial, age, sex and study phase as an interaction term, history of diabetes mellitus, ischemic heart disease or cerebrovascular disease, high sensitivity cardiac troponin I, creatinine concentration, and social deprivation. **(Middle)** Treatment commenced during index hospitalization in all patients and stratified by sex. **(Bottom)** Pre-existing treatment prior to index presentation in all patients and stratified by sex. ACE = angiotensin-converting enzyme; ARB = angiotensin receptor blocker; CI = confidence interval; DAPT = dual-antiplatelet therapy.

On comparing rates of the primary outcome between participants receiving different treatments, we observed heterogeneity between women and men (Figure 2). Women who received angiotensin-converting enzyme or angiotensin receptor blockers, statins, and beta-blockers had a similar reduction in risk for subsequent myocardial infarction or death from cardiovascular death as men. However, the efficacy was lower in women compared with men for coronary revascularization (adjusted odds ratio: 0.90 [95% confidence interval (CI): 0.68 to 1.17] vs. 0.51 [95% CI: 0.41 to 0.63]; p for interaction < 0.001) and the use of dual-antiplatelet therapy (adjusted odds ratio: 0.61 [95% CI: 0.49 to 0.76] vs. 0.42 [95% CI: 0.34 to 0.51]; p for interaction = 0.003) (Online Tables 6 and 7). Differences in treatment efficacy between women and men persisted in a sensitivity analysis restricted to those with type 1 myocardial infarction and were more pronounced in younger women compared with younger men for both revascularization and dual-antiplatelet therapy (p for interaction < 0.001 for both) (Online Tables 8 and 9, Online Figures 2 and 3).

### Clinical outcomes of women and men

**Figure 3:**
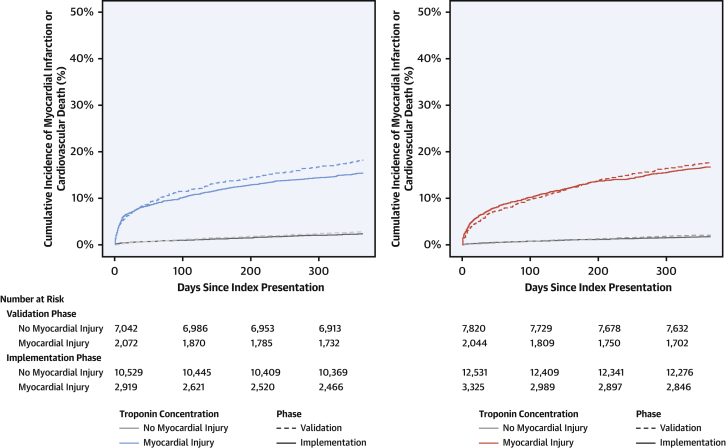
Incidence of Myocardial Infarction or Cardiovascular Death at 1 Year in Women and Men, Stratified by Troponin Concentration and Study Phase **(Left)** Cumulative incidence time-to-event curves for the primary outcome of myocardial infarction or cardiovascular death at 1 year for men admitted during the validation phase **(dashed line)** and implementation phase **(solid line)**. Patients are grouped according to whether myocardial injury was present **(blue)** or absent **(gray)**. Paired log-rank test results are p = 0.01 for men with myocardial injury and p = 0.06 for men without myocardial injury. **(Right)** Cumulative incidence time-to-event curves for the primary outcome of myocardial infarction or cardiovascular death at 1 year for women admitted during the validation phase **(dashed line)** and implementation phase **(solid line)**. Patients are grouped according to whether myocardial injury was present **(red)** or absent **(gray)**. Paired log-rank test results are p = 0.40 for women with myocardial injury and p = 0.08 for women without myocardial injury.

Overall, the primary outcome occurred in 5% of all patients (2,586 of 48,282) across both phases of the trial. The primary outcome occurred in 18% of women (369 of 2,072) with myocardial injury during the validation phase and in 17% (488 of 2,919) during the implementation phase (adjusted hazard ratio [HR]: 1.11; 95% CI: 0.92 to 1.33; p = 0.289) (Figure 3, Online Table 10). In men with myocardial injury, the primary outcome occurred in 18% (370 of 2,044) during the validation phase and in 15% (513 of 3,325) during the implementation phase (adjusted HR: 0.85; 95% CI: 0.71 to 1.01; p = 0.071; p for phase-sex interaction = 0.101) (Online Table 11). Primary and secondary outcomes in those women and men with type 1 myocardial infarction and reclassified by hs-cTnI are reported in Online Tables 12 and 13, respectively. Across both phases, there were no differences in the primary outcome between women and men already identified with myocardial injury (17% [857 of 4,991] vs. 16% [883 of 5,369]; HR: 0.88; 95% CI: 0.76 to 1.02), between women and men reclassified by the hs-cTnI assay (13% [195 of 1,470] vs. 14% [41 of 301]; HR: 0.93; 95% CI: 0.52 to 1.68), and between women and men without myocardial injury (2% [333 of 17,571] vs. 3% [513 of 20,351]; HR: 1.02; 95% CI: 0.82 to 1.27).

### Stratification by uniform diagnostic threshold

Women with hs-cTnI concentrations between the sex-specific threshold (16 ng/l) and the uniform threshold (26 ng/l) were of a similar age and had similar prevalence of pre-existing medical conditions and cardiovascular risk factors as women with hs-cTnI concentrations higher than 26 ng/l but were less likely to receive evidence-based treatment (Online Tables 14 and 15). Compared with women with hs-cTnI concentrations higher than 26 ng/l, women with hs-cTnI concentrations between 16 and 26 ng/l had similar index diagnoses and rates of the primary outcome at 1 year (13.4% [121 of 901] vs. 13.0% [74 of 569]) (Online Table 16).

## Discussion

The implementation of sex-specific thresholds for hs-cTnI had several important implications for the diagnosis, management, and outcomes of women and men with suspected acute coronary syndrome. First, it reclassified 5 times more additional women than men with myocardial injury. As such, the same proportion of women and men are now identified as having myocardial injury. Second, implementation was associated with increased rates of coronary angiography and revascularization, and increases in the use of secondary preventive therapy in all patients. Third, despite the identification of more women with myocardial injury following the adoption of sex-specific thresholds, women admitted during the implementation phase remained less likely to be investigated and treated for coronary artery disease than men. Finally, the rates of subsequent myocardial infarction or cardiovascular death were unchanged in both women and men following implementation of hs-cTnI testing (Central Illustration).

**Central Illustration:**
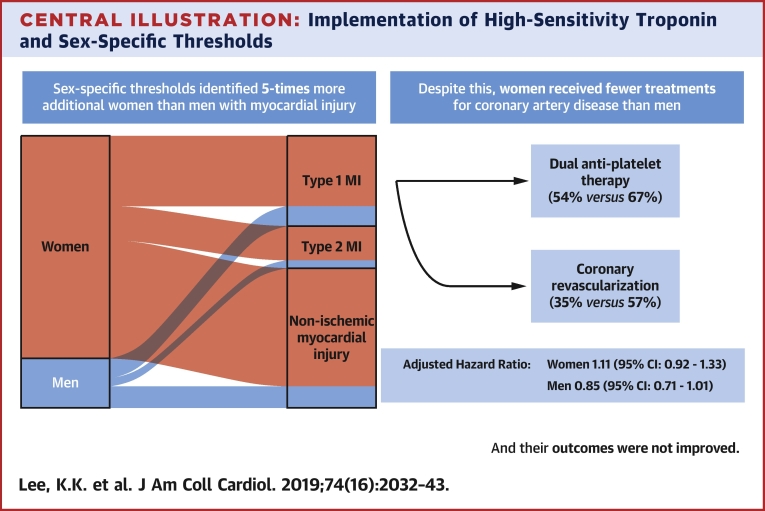
Implementation of High-Sensitivity Troponin and Sex-Specific Thresholds Sex-specific thresholds identified 5 times more additional women than men with myocardial injury. Despite this, women received fewer treatments for coronary artery disease than men, and their outcomes were not improved. CI = confidence interval; MI = myocardial infarction.

Despite the latest recommendations from the fourth universal definition of myocardial infarction, the majority of health care systems worldwide use a single threshold for the diagnosis of myocardial infarction in women and men (15). The impact of sex-specific thresholds on the diagnosis of myocardial infarction has been evaluated in a number of observational studies with divergent findings 7, 16, 17, 18, 19, 20. Most of these studies enrolled selected patients with acute coronary syndrome, of whom the majority were men. Furthermore, sex-specific thresholds were not used to guide clinical care or subsequent investigation for coronary artery disease. Here, we implemented sex-specific thresholds into routine clinical care in a randomized controlled trial and evaluated their impact in consecutive patients presenting with suspected acute coronary syndrome. We found that use of sex-specific thresholds identified proportionately more women, such that the overall percentages of women and men identified as having myocardial injury are now similar. This finding is intuitive, given that similar proportions of women and men had risk factors for ischemic heart disease, presented with chest pain, and had ischemic changes on electrocardiography. Implementation of sex-specific thresholds significantly increased the diagnosis of type 1 and type 2 myocardial infarction in women but had little impact in men.

Although hs-cTnI testing identified similar proportions of women and men with myocardial injury, we continue to observe major differences in their management. Women remain one-half as likely as men to receive treatment for acute coronary syndrome even after implementation of the hs-cTnI assay and sex-specific thresholds. A higher proportion of women had non-ischemic myocardial injury compared with men, and this might have accounted for the lower provision of therapies, because these therapies may not be indicated and there is no evidence from randomized controlled trials to guide treatment in this group of patients. However, this is not the only explanation for the disparity in treatment, as in our subgroup analyses restricted to those women and men with adjudicated diagnoses of type 1 myocardial infarction, treatment differences persisted.

Could differences in the provision and effectiveness of treatment explain why we did not observe an improvement in outcomes following the implementation of hs-cTnI? The primary analysis of the High-STEACS trial demonstrated no reduction in subsequent incidence of myocardial infarction or cardiovascular death at 1 year (8). Here, we report the primary outcome stratified by sex. The vast majority of patients reclassified by the sex-specific thresholds were women, but we did not observe any improvement in their outcomes. There was also no difference in the primary outcome in men, but as very few men were reclassified, we were underpowered for this subgroup.

A number of observations could explain our findings. First and perhaps most important, women were less likely to receive treatment for acute coronary syndrome than men. This was a consistent finding across both phases of the trial and in those with adjudicated diagnoses of type 1 myocardial infarction. The majority of patients reclassified by the hs-cTnI assay were women, who were less likely to receive evidence-based treatments than those already identified by the cTnI assay. This suggests that clinicians may be less willing to investigate or initiate treatment in patients with modest elevations in cardiac troponin. Second, we observed lower treatment efficacy for acute coronary syndrome in women compared with men. Third, women were older than men at presentation and more likely to die of noncardiovascular causes. It is possible that the opportunity to modify risk in this group of patients is more limited. However, it is unlikely that the lower provision and efficacy of treatment in women could be attributed to their older age at presentation alone. In a post hoc analysis, we observed that women younger than the median age were less likely to receive treatment than men of the same age. Furthermore, the observed differences in treatment efficacy between women and men were restricted to these younger women. It is plausible that the discordance in treatment provision and efficacy was due to differences in the pathophysiology of acute coronary syndromes between women and men. In observational studies, women are more likely than men to have plaque erosions and coronary microembolization (21), spontaneous coronary artery dissections (22), and coronary microvascular dysfunction (23), and women are consistently underrepresented in clinical trials of cardiovascular therapies 24, 25, 26. Women may also have been subjected to clinician bias in the use of diagnostic tests or provision of therapies (27) or have had a disproportionate burden of psychosocial risk factors, such as depression or a lack of social support, which can influence subsequent cardiovascular risk (28).

Should international guidelines recommend the use of sex-specific thresholds for the diagnosis of myocardial infarction? Although we did not directly compare a uniform 99th centile threshold against the sex-specific threshold in this trial, our study does provide some helpful insights into the use of sex-specific thresholds. First, use of sex-specific thresholds identifies similar proportions of women and men presenting with suspected acute coronary syndrome with myocardial injury. Second, women and men reclassified by the high-sensitivity assay with sex-specific thresholds were at similar risk for subsequent myocardial infarction or cardiovascular death. Therefore, use of a lower 99th centile in women appropriately identified a group of women who were at risk for cardiovascular events. Conversely, women and men with hs-cTnI concentrations below their respective sex-specific diagnostic thresholds had similar rates of cardiovascular events, suggesting that the use of a higher 99th centile for men did not inappropriately exclude myocardial injury in those who may have benefited from being identified and treated.

### Study limitations

First, it is possible that our adjudication panel may have underdiagnosed type 1 myocardial infarction in women, as the rates of coronary angiography were one-half those of men. We must acknowledge that some misclassification may have occurred.

Second, our estimates of treatment efficacy are subject to confounding by indication. Despite optimal adjustment, patients selected for treatment are likely to differ substantially from those who did not receive treatment, and we are unable to adjust for this residual confounding.

Third, the impact of adopting sex-specific thresholds will differ at hospitals that use lower uniform thresholds for cardiac troponin, as fewer women would be reclassified following implementation of hs-cTnI into practice.

Fourth, we have implemented sex-specific 99th centile thresholds using a high-sensitivity troponin I assay in our trial, but there are many other high-sensitivity assays in clinical use worldwide. All high-sensitivity cardiac troponin assays have demonstrated differences in the normal reference range between women and men, suggesting that this approach should be recommended for all assays (6). However, the impact of implementing sex-specific diagnostic thresholds will differ for those assays for which the difference between women and men is modest.

Fifth, our study population consists of patients who received troponin testing for suspected acute coronary syndrome in Scotland. We acknowledge that troponin testing may be performed for other reasons and varies across different health care systems 29, 30. The impact of implementing high-sensitivity cardiac troponin testing is likely to differ when used in a less selected patient population and when introduced at hospitals that have used either higher or lower cardiac troponin thresholds than used here.

Finally, we did not directly compare whether the use of sex-specific thresholds was superior to a uniform threshold using a hs-cTnI assay. This question is being evaluated in a prospective cluster randomized controlled trial (CODE-MI [Hs-cTn—Optimizing the Diagnosis of Acute Myocardial Infarction/Injury in Women] trial; NCT03819894) that will include a health economic analysis to determine whether adopting sex-specific thresholds and identifying more women at risk is cost effective.

## Conclusions

We report that use of a hs-cTnI assay with sex-specific thresholds identified 5 times as many additional women with myocardial injury than men, such that the proportion of women and men with myocardial injury is now equivalent. Despite this increase, women remain less likely than men to receive treatment for myocardial infarction, and the rates of subsequent myocardial infarction or cardiovascular death were not substantially reduced in either women or men following the implementation of high-sensitivity troponin testing into clinical practice.

Perspectives **COMPETENCY IN PATIENT CARE AND PROCEDURAL SKILLS:** A sex-specific threshold level of high-sensitivity cardiac troponin identifies a higher proportion of women with myocardial injury and infarction but may not improve clinical outcomes when there are disparities in treatment implementation or efficacy.

**TRANSLATIONAL OUTLOOK:** Future studies should focus on improving access to angiographic evaluation and therapeutic interventions for women with acute coronary syndromes.
