# Supplementary material for: Sex-Specific Thresholds of High-Sensitivity Troponin in Patients With Suspected Acute Coronary Syndrome
Source: J Am Coll Cardiol. 2019 Oct 22;74(16):2032–43. doi: 10.1016/j.jacc.2019.07.082 (PMC6876271; doi:10.1016/j.jacc.2019.07.082)
Supplement: Online Data [file mmc1.docx]

##### data supplement

Sex-Specific Thresholds of High-Sensitivity Troponin in Patients With Suspected Acute Coronary Syndrome

Kuan Ken Lee, M.D.,^1^* Amy V. Ferry, M.Sc.,^1^* Atul Anand, M.D,^1^ Fiona E. Strachan, Ph.D.,^1^ Andrew R. Chapman, M.D.,^1^ Dorien M. Kimenai, Ph.D.,^2,3^ Steven J.R. Meex, Ph.D.,^2,3^ Colin Berry, M.D.,^4^ Iain Findlay, M.D.,^5^ Alan Reid, M.Sc.,^6^ Anne Cruickshank, M.D.,^6^ Alasdair Gray, M.D.,^7^ Paul O. Collinson, M.D.,^8^ Fred S. Apple, Ph.D.,^9^ David A. McAllister, M.D.,^10^ Donogh Maguire, M.D.,^11^ Keith A.A. Fox, M.B.Ch.B.,^1^ David E. Newby, M.D.,^1^ Chris Tuck, B.Sc.,^12^ Catriona Keerie, M.Sc.,^12^ Christopher J. Weir, Ph.D.,^12^ Anoop S.V. Shah, M.D.,^1, 13^* Nicholas L. Mills, M.D.^1, 13^*;

*on behalf of the High-STEACS Investigators*^†^

^1^ BHF Centre for Cardiovascular Science, University of Edinburgh, Edinburgh, UK.

^2^ CARIM School for Cardiovascular Diseases, Maastricht University, Maastricht, the Netherlands

^3^ Central Diagnostic Laboratory, Maastricht University Medical Center, Maastricht, The Netherlands

^4^ Institute of Cardiovascular and Medical Sciences, University of Glasgow, Glasgow, UK.

^5^ Department of Cardiology, Royal Alexandra Hospital, Paisley, UK.

^6^ Department of Biochemistry, Queen Elizabeth University Hospital, Glasgow, UK.

^7^ Emergency Medicine Research Group Edinburgh, Royal Infirmary of Edinburgh, Edinburgh, UK.

^8^ Departments of Clinical Blood Sciences and Cardiology, St George’s, University Hospitals NHS Trust and St George’s University of London, London, UK.

^9^ Department of Laboratory Medicine and Pathology, Hennepin County Medical Center & University of Minnesota, Minneapolis, MN, USA

^10^ Institute of Health and Wellbeing, University of Glasgow, Glasgow, UK.

^11^ Emergency Medicine Department, Glasgow Royal Infirmary, Glasgow, UK.

^12^ Edinburgh Clinical Trials Unit, University of Edinburgh, Edinburgh, UK.

^13^ Usher Institute of Population Health Sciences and Informatics, University of Edinburgh, Edinburgh, UK.

*Contributed equally

^†^Listed at the end of the manuscript

**Corresponding Author:**

Professor Nicholas L Mills

BHF/University Centre for Cardiovascular Science

The University of Edinburgh

Edinburgh EH16 4SA

United Kingdom

Telephone: 0044 131 242 6515

E-mail: [nick.mills@ed.ac.uk](mailto:nick.mills@ed.ac.uk)

**Online Figures & Tables:** 19

**Randomization**

Block randomization was used with sites paired based on the expected number of presentations (***Online Table 1***) and one site randomized to early implementation and the other to late implementation. For pragmatic reasons (shared lab facilities out of hours), the Vale of Leven and Royal Alexandra Hospital, Paisley were grouped and randomized together. This enabled implementation of the high-sensitivity assay to occur on the same date at both sites and allowed the same lab processes to be followed at both sites. The randomization sequence was generated by a programmer at the Edinburgh Clinical Trials Unit who was not otherwise involved in the study using computer generated pseudo-random numbers.

**Implementation support**

To support implementation, we provided written educational material and presentations at each site, training for clinical and laboratory staff, and we updated the electronic patient record to highlight the change in assay and diagnostic thresholds. Educational material on the new assay and decision thresholds was presented at each Emergency Department handover (twice daily) during the implementation phase to ensure wide coverage of staff on all shift patterns. This was reinforced by specialist chest pain nurses who received detailed training prior to implementation and who support Emergency Department clinicians in the assessment of patients with suspected acute coronary syndrome. Key details from the educational presentation formed a one-page reference guide that was posted within each department and online in the hospital guidelines portal. This information was also presented to the wider hospital teams in medical grand round presentations prior to implementation, and circulated to all general practitioners. Every high-sensitivity cardiac troponin result reported in the electronic health record during the implementation phase was accompanied with guidance notes outlining the new assay, reporting units and thresholds. Laboratory staff also received training to ensure any queries directed to the laboratory were dealt with consistently. Finally, the research team included senior cardiologists, emergency physicians, and cardiology nurses who are clinically active within each of the hospital clusters; education was therefore reinforced at a local level by these clinical leaders throughout the implementation phase.

**Adjudication of the diagnosis of myocardial infarction**

Type 1 myocardial infarction was defined as myocardial necrosis (any hs-cTnI concentration above the sex-specific 99th centile with a rise and/or fall in hs-cTnI concentration where serial testing was performed) in the context of a presentation with suspected acute coronary syndrome with symptoms or signs of myocardial ischemia on the electrocardiogram. Type 2 myocardial infarction was defined as myocardial necrosis with symptoms or signs of myocardial ischemia due to increased oxygen demand or decreased oxygen supply secondary to an alternative pathology such as tachyarrhythmia, hypotension or anemia. Type 4b myocardial infarction was defined as myocardial injury with symptoms or signs of myocardial ischemia secondary to stent thrombosis demonstrated on coronary angiography. Patients with hs-cTnI concentrations above the 99^th^ centile without symptoms or signs of myocardial ischemia were classified as having non-ischemic myocardial injury.

**Outcomes**

All in-hospital and community deaths, and all hospital admissions are recorded on the Register of Deaths in Scotland and the Scottish Morbidity Record (SMR) respectively. It is a statutory requirement that any deaths occurring in Scotland, or outwith Scotland but within the United Kingdom are entered on the Register of Deaths in Scotland within eight days of death. As such, this registry is 100% complete for the study population, which was restricted to those resident in Scotland. This makes an assumption that patients did not emigrate in the year following enrollment. However, the Scottish population is very stable, with low levels of emigration outwith the United Kingdom.

The TrakCare software application (InterSystems Corporation, Cambridge, MA, USA) is an electronic patient record system used at all participating sites, which provided clinical data for all subsequent hospital admissions. All attendances across any participating hospital where cardiac troponin was measured and the hs-cTnI concentration was >99^th^ centile were reviewed and the diagnosis adjudicated. We used the same approach to adjudication as for the index hospital episode with the panel blinded to all cardiac troponin measurements during the index episode and to the study phase.

The primary outcome was myocardial infarction (type 1 or type 4b) or cardiovascular death at 1 year. Secondary efficacy end-points include myocardial infarction, unplanned coronary revascularisation, cardiovascular death, cardiac death, all-cause death, duration of stay, hospitalisation for heart failure, and ischemic stroke. Secondary safety end-points include major hemorrhage, unplanned hospitalisation excluding acute coronary syndrome, and non-cardiovascular death.

Unplanned coronary revascularisation was defined as any urgent or emergency percutaneous coronary intervention or coronary artery bypass grafting following discharge. International Classification of Disease (ICD)-10 codes from the Scottish Morbidity Record were used to define hospitalisation for heart failure (I50) and ischemic stroke (I63, I65, or I66). Bleeding was defined according to the Bleeding Academic Research Consortium (BARC) definition using ICD-10 and OPCS codes to classify each bleeding event as previously described.^1,2^ Major hemorrhage was defined as BARC type 3 or type 5. Unplanned hospitalisation excluding acute coronary syndrome was defined as any hospital attendance or admission excluding type 1 or type 4b myocardial infarction at 30 days.

The duration of stay was derived from a common electronic patient record system used across all participating sites (TrakCare, InterSystems Corporation, Cambridge, MA, USA) and was calculated from the admission and discharge date and time to the nearest minute.

The decision to perform coronary angiography was made by the attending cardiologist taking into consideration all aspects of the patients’ presentation including cardiac troponin concentrations.

The Scottish national community drug-prescribing database of ISD in NHS Scotland maintains a detailed record of all prescriptions dispensed in the community, which are linked to individual patient identifiers. Alterations in cardiovascular therapies following the index hospital episode were determined by comparison to baseline.

The Scottish Index of Multiple Deprivation (SIMD) identifies areas where the highest concentration of deprivation exists in Scotland. SIMD 2012 combines 38 different indicators covering seven different dimensions of deprivation including income, employment, health, education, housing, access to services and crime.^3^ The SIMD is derived for each individual in the trial population from the postcode at their address of residence.

We calculated Global Registry of Acute Coronary Events (GRACE) risk scores and stratified patients as low risk (<1% risk of in-hospital mortality) or intermediate–high risk (≥1% risk of in-hospital mortality.^4^

**Reporting**

We report all primary and secondary efficacy outcomes in the pre-specified statistical analysis plan. However, two of the eleven secondary end-points specified in the original trial protocol are not reported. During the conduct of the trial it became clear that ‘minor haemorrhage’ and ‘cardiovascular death excluding acute coronary syndrome’ would be difficult to define from routine healthcare data. Following discussion with our trial steering committee, these secondary endpoints were not included in the pre-specified statistical analysis plan and are therefore not presented in the manuscript.

**Site Closures**

In order to accommodate the closure of the Western Infirmary and Victoria Infirmary during the implementation phase of the study, and subsequent redirection of patients in their catchment areas to the new Queen Elizabeth University Hospital on the site of the Southern General Hospital, patients were analysed according to the site at which they were treated. The Queen Elizabeth University Hospital was considered a continuation of the Southern General Hospital site.

**Sample Size**

Using previous data from the Royal Infirmary of Edinburgh,^5^ we estimated that patients reclassified by the high-sensitivity assay would experience an event rate of 13% for the primary outcome of subsequent myocardial infarction or cardiovascular death. We originally planned that 10 sites (clusters) would include patients during three 6-month phases: validation (standard care), randomization (early or late introduction of the intervention) and implementation (intervention). For each site the difference in proportions of primary outcome events between standard care and intervention will be approximately normally distributed, each with a standard deviation that depends on the number of patients recruited by that site and the primary outcome event rate under standard care (assumed to be 13%). During a pilot phase,^6^ detailed power calculations, based on 1,000 simulations per scenario, were performed based on the anticipated proportion of patients who would be reclassified by the high-sensitivity assay. Power for a reclassification rate ranging from 6% to 9%, was 74% to 85% for an absolute risk reduction of 4·4%. Power was virtually unchanged when varying the ICC value from 0·05 to 0·10, as would be expected in a stepped wedge design such as this in which relatively few clusters recruit a large number of patients per site.

**Statistical analyses**

The primary outcome was compared before and after implementation of the hs-cTnI assay stratified by sex in those with myocardial injury using a Cox proportional hazards model. We used the same model to compare the primary outcome in women and men reclassified by the hs-cTnI assay and in post-hoc analyses. This model included all variables from the primary analysis of the HighSTEACS trial.^7^ However, as women were significantly older and had more comorbidities than men, we included additional adjustment for comorbidities here, in accordance with the STROBE guidelines.^8^ The model adjusted for hospital site (fitted as a random effect), season, time of presentation from the start date of the trial, age, sex and study phase as an interaction term, previous history of diabetes mellitus, ischemic heart disease or cerebrovascular disease, hs-cTnI, creatinine concentrations and social deprivation.

In a post-hoc analysis, we used the same Cox proportional hazards model to compare the primary outcome between women and men already identified with myocardial injury by the cTnI assay, between women and men reclassified by the hs-cTnI assay and between women and men without myocardial injury across both study phases. Furthermore, we evaluated treatment efficacy in logistic regression models comparing the primary outcome in those who received treatment versus those who did not in all patients and stratified by sex. Logistic regression models were adjusted for the same variables as the primary cox proportional hazards model. We performed sensitivity analyses of treatment efficacy in patients with type 1 myocardial infarction and stratified by the median age of all patients with myocardial injury. All statistical analyses were performed using R Version 3.5

**Data Sharing**

The High-STEACS trial makes use of multiple routine electronic health care data sources that are linked, deidentified and held in our national safe haven, which is accessible by approved individuals who have undertaken the necessary governance training. Summary data can be made available upon request to Nicholas Mills ([nick.mills@ed.ac.uk](mailto:nick.mills@ed.ac.uk)).

**The High-STEACS Investigators**

**Chief Investigator:** Prof Nicholas L Mills.

**Trial managers:** Dr Fiona E Strachan and Mr Christopher Tuck.

**Trial research team:** Dr Anoop SV Shah, Dr Atul Anand, Ms Amy V Ferry, Dr Kuan Ken Lee, Dr Andrew R Chapman, Mr Dennis Sandeman, Dr Philip D Adamson, Dr Catherine L Stables, Dr Catalina A Vallejo, Dr Athanasios Tsanasis, Ms Lucy Marshall, Ms Stacey D Stewart, Dr Takeshi Fujisawa, Ms Mischa Hautvast, Ms Jean McPherson and Ms Lynn McKinlay.

**Grant applicants:** Prof Nicholas L Mills (Principal Applicant), Prof David E Newby, Prof Keith AA Fox, Prof Colin Berry, Dr Simon Walker, and Dr Christopher J Weir.

**Trial steering committee:** Prof Ian Ford (chair, independent), Prof Nicholas L Mills, Prof David E Newby, Prof Alasdair Gray, Prof Keith AA Fox, Prof Colin Berry, Dr Simon Walker, Prof Paul O Collinson, Prof Fred S Apple, Mr Alan Reid, Dr Anne Cruikshank, Dr Iain Findlay, Dr Shannon Amoils (independent), Dr David A McAllister, Dr Donogh Maguire, Ms Jennifer Stevens (independent), Prof John Norrie (independent), and Prof Christopher Weir.

**Adjudication panel:** Dr Anoop SV Shah, Dr Atul Anand, Dr Andrew R Chapman, Dr Kuan Ken Lee, Dr Jack PM Andrews, Dr Philip D Adamson, Dr Alastair Moss, Dr Mohamed S Anwar, Dr John Hung, Prof Nicholas L Mills.

**Biochemistry sub-group committee:** Dr Simon Walker, Dr Jonathan Malo, Mr Alan Reid, Dr Anne Cruikshank, Prof Paul O Collinson.

**Data monitoring committee:** Prof Colin M Fischbacher, Dr Bernard L Croal, Prof Stephen J Leslie.

**Edinburgh Clinical Trials Unit:** Ms Catriona Keerie, Mr Richard A Parker, Mr Allan Walker, Mr Ronnie Harkess, Mr Christopher Tuck, Mr Tony Wackett, Prof Christopher Weir.

**NHS Greater Glasgow & Clyde Safe Haven:** Dr Roma Armstrong, Ms Marion Flood, Ms Laura Stirling, Ms Claire MacDonald, Mr Imran Sadat, Mr Frank Finlay.

**NHS Lothian Research Governance, eHealth and Safe Haven:** Dr Heather Charles, Ms Pamela Linksted, Mr Stephen Young, Mr Bill Alexander, Mr Chris Duncan.

**Online Table 1: Number of Hospitalizations with Chest Pain and Myocardial Infarction at Participating Sites in 2010**

|  | Chest pain | Myocardial infarction | Assay platform available | Diagnostic threshold for contemporary cTnI assay |
| --- | --- | --- | --- | --- |
| Vale of Leven General Hospital | 455 | 132 | Yes | 40 ng/L |
| Inverclyde Royal Hospital | 980 | 135 | Yes | 40 ng/L |
| Royal Alexandra Hospital | 1,371 | 178 | Yes | 40 ng/L |
| Glasgow Royal Infirmary | 1,791 | 227 | Yes | 40 ng/L |
| Victoria Infirmary | 1,670 | 315 | Yes | 40 ng/L |
| Southern General Hospital | 1,274 | 163 | Yes | 40 ng/L |
| Western Infirmary | 1,867 | 187 | Yes | 40 ng/L |
| Western General Hospital | 379 | 103 | Yes | 50 ng/L |
| St John’s Hospital | 1,001 | 211 | Yes | 50 ng/L |
| Royal Infirmary of Edinburgh | 2,051 | 864 | Yes | 50 ng/L |
| *Source: Scottish Morbidity Record from eDRIS NHS Scotland for 2010 linked on 26 February 2011 using the following ICD-10 codes: chest pain (R7) and myocardial infarction (I21, I22). cTnI = cardiac troponin I* | | | | |

**Online Table 2: Characteristics of trial participants, stratified by troponin concentration and gender**

|  | No myocardial injury | | | Reclassified by high-sensitivity cardiac troponin I assay | | | Identified by cardiac troponin I assay | | | |
| --- | --- | --- | --- | --- | --- | --- | --- | --- | --- | --- |
|  | **Overall** | **Women** | **Men** | **Overall** | **Women** | **Men** | **Overall** | **Women** | **Men** |  |
| Number of participants | 37922 | 17571 | 20351 | 1771 | 1470 | 301 | 8589 | 3521 | 5068 |  |
| Age, years | 58 (17) | 60 (17) | 57 (17) | 75 (14) | 76 (13) | 70 (15) | 70 (15) | 74 (14) | 68 (15) |  |
| Phase |  |  |  |  |  |  |  |  |  |  |
| Validation | 14862 (39) | 7042 (40) | 7820 (38) | 720 (41) | 612 (42) | 108 (36) | 3396 (40) | 1460 (42) | 1936 (38) |  |
| Implementation | 23060 (61) | 10529 (60) | 12531 (62) | 1051 (59) | 858 (58) | 193 (64) | 5193 (61) | 2061 (59) | 3132 (62) |  |
| Presenting complaint* |  |  |  |  |  |  |  |  |  |  |
| Chest pain | 28091 (84) | 12896 (84) | 15195 (84) | 1074 (67) | 869 (66) | 205 (71) | 5375 (71) | 2011 (65) | 3364 (74) |  |
| Dyspnoea | 1107 (3) | 507 (3) | 600 (3) | 202 (13) | 161 (12) | 41 (14) | 866 (11) | 414 (14) | 452 (10) |  |
| Palpitation | 991 (3) | 550 (4) | 441 (2) | 72 (5) | 64 (5) | 8 (3) | 206 (3) | 108 (4) | 98 (2) |  |
| Syncope | 1809 (5) | 754 (5) | 1055 (6) | 125 (8) | 103 (8) | 22 (8) | 561 (7) | 285 (9) | 276 (6) |  |
| Other | 1458 (4) | 670 (4) | 788 (4) | 128 (8) | 115 (9) | 13 (5) | 602 (8) | 259 (8) | 343 (8) |  |
| Previous medical conditions |  |  |  |  |  |  |  |  |  |  |
| Myocardial infarction | 2835 (8) | 995 (6) | 1840 (9) | 219 (12) | 164 (11) | 55 (18) | 1160 (14) | 467 (13) | 693 (14) |  |
| Ischemic heart disease | 8455 (22) | 3429 (20) | 5026 (25) | 645 (36) | 526 (36) | 119 (40) | 2812 (33) | 1137 (32) | 1675 (33) |  |
| Cerebrovascular disease | 1915 (5) | 857 (5) | 1058 (5) | 210 (12) | 178 (12) | 32 (11) | 824 (10) | 395 (11) | 429 (9) |  |
| Diabetes mellitus | 2040 (5) | 785 (5) | 1255 (6) | 218 (12) | 163 (11) | 55 (18) | 1260 (15) | 501 (14) | 759 (15) |  |
| Previous revascularisation |  |  |  |  |  |  |  |  |  |  |
| PCI | 2744 (7) | 835 (5) | 1909 (9) | 155 (9) | 114 (8) | 41 (14) | 783 (9) | 241 (7) | 542 (11) |  |
| CABG | 534 (1) | 124 (1) | 410 (2) | 40 (2) | 24 (2) | 16 (5) | 208 (2) | 52 (2) | 156 (3) |  |
| Medications at presentation |  |  |  |  |  |  |  |  |  |  |
| Aspirin | 9462 (25) | 3940 (22) | 5522 (27) | 668 (38) | 555 (38) | 113 (38) | 3033 (35) | 1204 (34) | 1829 (36) |  |
| P2Y12 inhibitor | 3162 (8) | 1354 (8) | 1808 (9) | 270 (15) | 215 (15) | 55 (18) | 1152 (13) | 535 (15) | 617 (12) |  |
| Dual anti-platelet therapy† | 1103 (3) | 382 (2) | 721 (4) | 88 (5) | 61 (4) | 27 (9) | 414 (5) | 167 (5) | 247 (5) |  |
| Statin | 14106 (37) | 6051 (34) | 8055 (40) | 960 (54) | 791 (54) | 169 (56) | 4300 (50) | 1708 (49) | 2592 (51) |  |
| ACE or ARB | 11285 (30) | 4845 (28) | 6440 (32) | 762 (43) | 614 (42) | 148 (49) | 3571 (42) | 1445 (41) | 2126 (42) |  |
| Beta-blocker | 9566 (25) | 4272 (24) | 5294 (26) | 658 (37) | 549 (37) | 109 (36) | 2949 (34) | 1260 (36) | 1689 (33) |  |
| Oral anticoagulant‡ | 2158 (6) | 962 (6) | 1196 (6) | 238 (13) | 203 (14) | 35 (12) | 857 (10) | 384 (11) | 473 (9) |  |
| Loop diuretic | 3737 (10) | 2015 (12) | 1722 (9) | 570 (32) | 506 (34) | 64 (21) | 2123 (25) | 1065 (30) | 1058 (21) |  |
| Proton pump inhibitor | 15434 (41) | 7733 (44) | 7701 (38) | 880 (50) | 742 (51) | 138 (46) | 3758 (44) | 1730 (49) | 2028 (40) |  |
| Calcium channel blocker | 5059 (13) | 2263 (13) | 2796 (14) | 329 (19) | 263 (18) | 66 (22) | 1648 (19) | 658 (19) | 990 (20) |  |
| Nicorandil | 1644 (4) | 681 (4) | 963 (5) | 100 (6) | 90 (6) | 10 (3) | 545 (6) | 213 (6) | 332 (7) |  |
| Ivabradine | 289 (0) | 118 (1) | 171 (1) | 30 (2) | 26 (2) | <5 | 116 (1) | 42 (1) | 74 (2) |  |
| Spironolactone | 634 (2) | 250 (1) | 384 (2) | 84 (5) | 67 (5) | 17 (6) | 366 (4) | 134 (4) | 232 (5) |  |
| Electrocardiogram result§ |  |  |  |  |  |  |  |  |  |  |
| Normal |  |  |  | 592 (43) | 498 (45) | 94 (36) | 2080 (32) | 868 (33) | 1212 (32) |  |
| Myocardial ischemia |  |  |  | 194 (14) | 151 (14) | 43 (17) | 2316 (36) | 872 (33) | 1444 (38) |  |
| ST segment elevation |  |  |  | 32 (2) | 23 (2) | 9 (4) | 966 (15) | 306 (12) | 660 (17) |  |
| ST segment depression |  |  |  | 125 (9) | 103 (9) | 22 (9) | 1203 (19) | 480 (18) | 723 (19) |  |
| T wave inversion |  |  |  | 192 (14) | 159 (14) | 33 (13) | 1085 (17) | 481 (18) | 604 (16) |  |
| Physiological parameters |  |  |  |  |  |  |  |  |  |  |
| Heart rate, beats per minute | 77 (25) | 74 (23) | 94 (33) | 86 (27) | 87 (27) | 82 (25) | 85 (26) | 89 (26) | 84 (26) |  |
| Systolic blood pressure, mmHg | 138 (23) | 136 (24) | 145 (19) | 143 (28) | 144 (28) | 141 (28) | 137 (29) | 140 (30) | 136 (28) |  |
| GRACE risk score | 124 (38) | 129 (31) | 108 (54) | 141 (32) | 143 (32) | 136 (35) | 143 (39) | 149 (38) | 140 (39) |  |
| Hematology and clinical chemistry measurements |  |  |  |  |  |  |  |  |  |  |
| Hemoglobin, g/L | 137 (20) | 130 (18) | 143 (20) | 126 (22) | 125 (22) | 134 (23) | 132 (26) | 125 (24) | 137 (25) |  |
| eGFR, ml/min | 56 (11) | 56 (11) | 56 (11) | 47 (15) | 47 (15) | 46 (16) | 48 (16) | 46 (16) | 49 (15) |  |
| Peak high-sensitivity cardiac troponin I, ng/L | 3 (1–6) | 2 (1–5) | 3 (2–7) | 26 (20–37) | 24 (20–31) | 41 (37–47) | 297 (76–2600) | 225 (70–1706) | 372 (80–3486) |  |
| Serial high-sensitivity cardiac troponin I, %¶ | 16028 (42) | 7275 (41) | 8753 (43) | 1024 (58) | 837 (57) | 187 (62) | 5959 (69) | 2393 (68) | 3566 (70) |  |
| Adjudicated diagnosis# |  |  |  |  |  |  |  |  |  |  |
| Type 1 MI |  |  |  | 515 (33) | 401 (32) | 114 (40) | 4513 (60) | 1609 (52) | 2904 (65) |  |
| Type 2 MI |  |  |  | 240 (16) | 195 (15) | 45 (16) | 1020 (14) | 505 (16) | 515 (12) |  |
| Non-ischemic myocardial injury |  |  |  | 796 (51) | 673 (53) | 123 (44) | 2014 (27) | 1000 (32) | 1014 (23) |  |

Presented as number of patients (%), mean (SD) or median (IQR). Abbreviations: ACE = angiotensin converting enzyme; ARB = angiotensin receptor blocker; eGFR = estimated glomerular filtration rate; PCI = percutaneous coronary intervention; CABG = coronary artery bypass grafting; UDMI = Universal Definition of Myocardial Infarction.

*A presenting symptom was missing in 5615 (12%) patients. †Two medications from aspirin, clopidogrel, prasugrel or ticagrelor. ‡Includes warfarin or novel oral anti-coagulants. §Electrocardiographic data was available in 1377 (78%) of reclassified patients and 6470 (75%) of identified patients. ¶Defined as two or more tests within 24 hours from presentation. #The adjudication panel were able to achieve a consensus diagnosis in 9,115 (88%) patients with hs-cTnI concentrations above the sex-specific 99th centile.

**Online Table 3: Management of patients during initial hospital admission, stratified by study phase and gender in patients reclassified by high-sensitivity cardiac troponin I assay**

|  | Overall | Women | | | Men | | |
| --- | --- | --- | --- | --- | --- | --- | --- |
|  |  | **Overall** | **Validation** | **Implementation** | **Overall** | **Validation** | **Implementation** |
| Number of participants | 1771 | 1470 | 612 | 858 | 301 | 108 | 193 |
| Duration of hospital stay, h | 34 (9–121) | 33 (7–122) | 21 (4–108) | 51 (20–135) | 38 (17–116) | 23 (4–87) | 47 (22–127) |
| Coronary angiography | 140 (8) | 100 (7) | 20 (3) | 80 (9) | 40 (13) | 9 (8) | 31 (16) |
| PCI | 70 (4) | 52 (4) | 16 (3) | 36 (4) | 18 (6) | 6 (6) | 12 (6) |
| CABG | <5 | <5 | <5 | <5 | <5 | <5 | <5 |
| PCI or CABG | 74 (4) | 53 (4) | 16 (3) | 37 (4) | 21 (7) | 7 (6) | 14 (7) |
| New anti-platelet drug | 258 (15) | 202 (14) | 55 (9) | 147 (17) | 56 (19) | 9 (8) | 47 (24) |
| New DAPT | 159 (9) | 121 (8) | 31 (5) | 90 (11) | 38 (13) | <5 | 34 (18) |
| New statin therapy | 111 (6) | 82 (6) | 24 (4) | 58 (7) | 29 (10) | 8 (7) | 21 (11) |
| New ACE or ARB | 111 (6) | 84 (6) | 26 (4) | 58 (7) | 27 (9) | 8 (7) | 19 (10) |
| New beta-blocker | 229 (13) | 184 (135) | 55 (9) | 129 (15) | 45 (15) | 10 (9) | 35 (18) |
| New oral anticoagulant | 124 (7) | 99 (7) | 34 (6) | 65 (8) | 25 (8) | 6 (6) | 19 (10) |

Presented as number of patients (%) or median (IQR). Abbreviations: ACE = angiotensin converting enzyme; ARB = angiotensin receptor blocker; DAPT= dual anti-platelet therapy; PCI = percutaneous coronary intervention; CABG = coronary artery bypass grafting.

**Online Table 4: Management of patients during the implementation phase, stratified by troponin concentration and sex.**

|  | Reclassified by high-sensitivity cardiac troponin I assay | | Identified by cardiac troponin I assay | |
| --- | --- | --- | --- | --- |
|  | Women | Men | Women | Men |
| Number of participants | 858 | 193 | 2061 | 3132 |
| Duration of hospital stay, h | 51 [19-135] | 47 [22-127] | 93 [42-194] | 73 [34-142] |
| Coronary angiography | 80 (9) | 31 (16) | 673 (33) | 1504 (48) |
| PCI | 36 (4) | 12 (6) | 394 (19) | 1054 (34) |
| CABG | <10 | <10 | 19 (1) | 71 (2) |
| PCI or CABG | 37 (4) | 14 (7) | 412 (20) | 1123 (36) |
| New anti-platelet drug | 147 (17) | 47 (24) | 838 (41) | 1590 (51) |
| New DAPT | 90 (11) | 34 (18) | 682 (33) | 1398 (45) |
| New statin therapy | 58 (7) | 21 (11) | 409 (20) | 854 (27) |
| New ACE or ARB | 58 (7) | 19 (10) | 372 (18) | 791 (25) |
| New beta-blocker | 129 (15) | 35 (18) | 509 (25) | 993 (32) |
| New oral anticoagulant | 65 (8) | 19 (10) | 129 (6) | 194 (6) |

*Presented as number of patients (%) or median (IQR). Abbreviations: ACE = angiotensin converting enzyme; ARB = angiotensin receptor blocker; DAPT= dual anti-platelet therapy; PCI = percutaneous coronary intervention; CABG = coronary artery bypass grafting.*

**Online Table 5: Management of patients with type 1 myocardial infarction during initial hospital admission, stratified by sex and study phase**

|  | Overall | Women | | | | Men | | | |
| --- | --- | --- | --- | --- | --- | --- | --- | --- | --- |
|  |  | **Overall** | **Validation** | **Implementation** | P value* | **Overall** | **Validation** | **Implementation** | P value* |
| Number of participants | 5028 | 2010 | 748 | 1262 |  | 3018 | 1059 | 1959 |  |
| Duration of hospital stay, hrs | 75 (41–126) | 78 (35–142) | 76 (25–151) | 78 (45–135) | 0.240 | 73 (43–119)† | 75 (27–122) | 73 (46–118) | 0.224 |
| Coronary angiography | 3117 (62) | 991 (49) | 321 (43) | 670 (53) | <0.001 | 2126 (70)† | 703 (66) | 1423 (73) | <0.001 |
| PCI | 2115 (42) | 609 (30) | 185 (25) | 424 (34) | <0.001 | 1506 (50)† | 459 (43) | 1047 (53) | <0.001 |
| CABG | 143 (3) | 27 (1) | 8 (1) | 19 (2) | 0.535 | 116 (4)† | 50 (5) | 66 (3) | 0.081 |
| PCI or CABG | 2253 (45) | 635 (32) | 193 (26) | 442 (35) | <0.001 | 1618 (54)† | 507 (48) | 1111 (57) | <0.001 |
| New anti-platelet drug | 3392 (67) | 1216 (60) | 430 (57) | 786 (62) | 0.038 | 2176 (72)† | 730 (69) | 1446 (74) | 0.005 |
| New DAPT | 3003 (60) | 1045 (52) | 358 (48) | 687 (54) | 0.005 | 1958 (65)† | 642 (61) | 1316 (67) | <0.001 |
| New statin therapy | 1771 (35) | 576 (29) | 182 (24) | 394 (31) | 0.001 | 1195 (40)† | 395 (37) | 800 (41) | 0.066 |
| New ACE or ARB | 1593 (32) | 513 (26) | 183 (24) | 330 (26) | 0.433 | 1080 (36)† | 383 (36) | 697 (36) | 0.764 |
| New beta-blocker | 1887 (38) | 616 (31) | 198 (26) | 418 (33) | 0.002 | 1271 (42)† | 447 (42) | 824 (42) | 0.952 |
| New oral anticoagulant | 130 (3) | 54 (3) | 21 (3) | 33 (3) | 0.908 | 76 (3) | 25 (2) | 51 (3) | 0.779 |

Presented as number of patients (%) or median (IQR). Abbreviations: ACE = angiotensin converting enzyme; ARB = angiotensin receptor blocker; DAPT= dual anti-platelet therapy; PCI = percutaneous coronary intervention; CABG = coronary artery bypass grafting.

^*^Comparison between validation and implementation phase. †P value <0.05 comparing women and men.

**Online Table 6: Logistic regression model, coefficients and standard errors for efficacy of coronary revascularization therapy in all patients with myocardial injury**

|  | **Estimate** | **Standard Error** | **z value** | **Pr (>\|z\|)** |
| --- | --- | --- | --- | --- |
| (Intercept) | -4.962 | 0.226 | -22 | <0.001 |
| Age | 0.033 | 0.002 | 13.722 | <0.001 |
| Log_2_ (Troponin) | 0.095 | 0.009 | 10.32 | <0.001 |
| Creatinine | 0.002 | 0.000 | 5.798 | <0.001 |
| Previous myocardial infarction | 0.348 | 0.086 | 4.042 | <0.001 |
| Previous ischemic heart disease | 0.27 | 0.071 | 3.827 | <0.001 |
| Previous cerebrovascular disease | 0.314 | 0.082 | 3.836 | <0.001 |
| Previous diabetes mellitus | 0.313 | 0.075 | 4.158 | <0.001 |
| Study phase | 0.05 | 0.074 | 0.681 | 0.496 |
| Frailty (study site) | -0.01 | 0.011 | -0.862 | 0.389 |
| Season: Summer | -0.076 | 0.083 | -0.916 | 0.36 |
| Season: Autumn | -0.166 | 0.084 | -1.98 | 0.048 |
| Season: Winter | -0.081 | 0.084 | -0.968 | 0.333 |
| Days since start of study | -0.003 | 0.001 | -2.211 | 0.027 |
| Deprivation quintile | 0.000 | 0.000 | -3.171 | 0.002 |
| Coronary revascularization | -0.144 | 0.13 | -1.111 | 0.267 |
| Sex | 0.137 | 0.063 | 2.165 | 0.03 |
| Coronary revascularization and sex interaction | -0.523 | 0.158 | -3.321 | <0.001 |

**Online Table 7: Logistic regression model, coefficients and standard errors for efficacy of dual antiplatelet therapy in all patients with myocardial injury**

|  | **Estimate** | **Standard Error** | **z value** | **Pr (>\|z\|)** |
| --- | --- | --- | --- | --- |
| (Intercept) | -4.811 | 0.225 | -21.424 | <0.001 |
| Age | 0.031 | 0.002 | 13.062 | <0.001 |
| Log_2_ (Troponin) | 0.107 | 0.009 | 11.57 | <0.001 |
| Creatinine | 0.001 | 0.000 | 5.116 | <0.001 |
| Previous myocardial infarction | 0.371 | 0.087 | 4.281 | <0.001 |
| Previous ischemic heart disease | 0.277 | 0.071 | 3.912 | <0.001 |
| Previous cerebrovascular disease | 0.292 | 0.082 | 3.558 | <0.001 |
| Previous diabetes mellitus | 0.335 | 0.076 | 4.412 | <0.001 |
| Study phase | 0.067 | 0.074 | 0.9 | 0.368 |
| Frailty (study site) | -0.009 | 0.011 | -0.751 | 0.453 |
| Season: Summer | -0.095 | 0.083 | -1.137 | 0.256 |
| Season: Autumn | -0.173 | 0.084 | -2.055 | 0.040 |
| Season: Winter | -0.123 | 0.084 | -1.468 | 0.142 |
| Days since start of study | -0.003 | 0.001 | -1.768 | 0.077 |
| Deprivation quintile | 0.000 | 0.000 | -3.161 | 0.002 |
| New dual antiplatelet therapy | -0.483 | 0.103 | -4.686 | <0.001 |
| Sex | 0.142 | 0.067 | 2.125 | 0.034 |
| New dual antiplatelet therapy and sex interaction | -0.394 | 0.133 | -2.966 | 0.003 |

**Online Table 8: Logistic regression model, coefficients and standard errors for efficacy of coronary revascularization therapy in patients with myocardial injury below the median age of 73 years**

|  | **Estimate** | **Standard Error** | **z value** | **Pr (>\|z\|)** |
| --- | --- | --- | --- | --- |
| (Intercept) | -4.356 | 0.382 | -11.409 | <0.001 |
| Age | 0.027 | 0.005 | 5.322 | <0.001 |
| Log_2_ (Troponin) | 0.049 | 0.015 | 3.217 | 0.001 |
| Creatinine | 0.001 | 0.000 | 3.286 | 0.001 |
| Previous myocardial infarction | 0.301 | 0.145 | 2.075 | 0.038 |
| Previous ischemic heart disease | 0.334 | 0.129 | 2.598 | 0.009 |
| Previous cerebrovascular disease | 0.465 | 0.153 | 3.038 | 0.002 |
| Previous diabetes mellitus | 0.415 | 0.117 | 3.54 | <0.001 |
| Study phase | 0.289 | 0.125 | 2.321 | 0.02 |
| Frailty (study site) | -0.006 | 0.019 | -0.319 | 0.75 |
| Season: Summer | -0.348 | 0.137 | -2.53 | 0.011 |
| Season: Autumn | -0.272 | 0.134 | -2.03 | 0.042 |
| Season: Winter | -0.193 | 0.135 | -1.425 | 0.154 |
| Days since start of study | 0.000 | 0.002 | -0.004 | 0.997 |
| Deprivation quintile | 0.000 | 0.000 | -2.882 | 0.004 |
| Coronary revascularization | 0.249 | 0.177 | 1.402 | 0.161 |
| Sex | 0.44 | 0.114 | 3.876 | <0.001 |
| Coronary revascularization and sex interaction | -0.932 | 0.211 | -4.414 | <0.001 |

**Online Table 9: Logistic regression model, coefficients and standard errors for efficacy of dual antiplatelet therapy in patients with myocardial injury below the median age of 73 years**

|  | **Estimate** | **Standard Error** | **z value** | **Pr (>\|z\|)** |
| --- | --- | --- | --- | --- |
| (Intercept) | -4.144 | 0.377 | -10.990 | <0.001 |
| Age | 0.025 | 0.005 | 4.978 | <0.001 |
| Log_2_ (Troponin) | 0.066 | 0.015 | 4.421 | <0.001 |
| Creatinine | 0.001 | 0.000 | 2.431 | 0.015 |
| Previous myocardial infarction | 0.348 | 0.147 | 2.373 | 0.018 |
| Previous ischemic heart disease | 0.301 | 0.130 | 2.326 | 0.020 |
| Previous cerebrovascular disease | 0.389 | 0.154 | 2.529 | 0.011 |
| Previous diabetes mellitus | 0.479 | 0.119 | 4.023 | <0.001 |
| Study phase | 0.303 | 0.125 | 2.431 | 0.015 |
| Frailty (study site) | -0.008 | 0.019 | -0.439 | 0.661 |
| Season: Summer | -0.390 | 0.139 | -2.817 | 0.005 |
| Season: Autumn | -0.290 | 0.135 | -2.147 | 0.032 |
| Season: Winter | -0.266 | 0.136 | -1.956 | 0.050 |
| Days since start of study | 0.001 | 0.002 | 0.214 | 0.831 |
| Deprivation quintile | 0.000 | 0.000 | -2.707 | 0.007 |
| New dual antiplatelet therapy | -0.317 | 0.169 | -1.875 | 0.061 |
| Sex | 0.472 | 0.119 | 3.960 | <0.001 |
| New dual antiplatelet therapy and sex interaction | -0.785 | 0.203 | -3.867 | <0.001 |

**Online Table 10: Primary and secondary outcomes after 1 year in participants with myocardial injury, stratified by sex and study phase**

|  | Overall | Women | | | Men | | |
| --- | --- | --- | --- | --- | --- | --- | --- |
|  |  | **Overall** | **Validation** | **Implementation** | **Overall** | **Validation** | **Implementation** |
| Number of participants | 10360 | 4991 | 2072 | 2919 | 5369 | 2044 | 3325 |
| Primary outcome |  |  |  |  |  |  |  |
| Myocardial infarction* or death from cardiovascular causes | 1740 (17) | 857 (17) | 369 (18) | 488 (17) | 883 (16) | 370 (18) | 513 (15) |
| Secondary outcomes |  |  |  |  |  |  |  |
| Myocardial infarction* | 685 (7) | 340 (7) | 156 (8) | 184 (6) | 345 (6) | 149 (7) | 196 (6) |
| Unplanned revascularisation† | 410 (4) | 150 (3) | 58 (3) | 92 (3) | 260 (5) | 107 (5) | 153 (5) |
| All-cause death | 2373 (23) | 1251 (25) | 566 (27) | 685 (24) | 1122 (21) | 483 (24) | 639 (19) |
| Death from cardiovascular causes | 1177 (11) | 575 (12) | 236 (11) | 339 (12) | 602 (11) | 250 (12) | 352 (11) |
| Death from cardiac causes | 939 (9) | 439 (9) | 172 (8) | 267 (9) | 500 (9) | 209 (10) | 291 (9) |
| Hospital admission for heart failure | 1029 (10) | 513 (10) | 229 (11) | 284 (10) | 516 (10) | 233 (11) | 283 (9) |
| Ischemic stroke | 202 (2) | 103 (2) | 51 (3) | 52 (2) | 99 (2) | 51 (3) | 48 (1) |
| Safety endpoints |  |  |  |  |  |  |  |
| Major hemorrhage‡ | 100 (1) | 46 (1) | 24 (1) | 22 (1) | 54 (1) | 19 (1) | 35 (1) |
| Unplanned hospital admission at 30 days§ | 3044 (29) | 1390 (28) | 645 (31) | 745 (26) | 1654 (31) | 753 (37) | 901 (27) |
| Non-cardiovascular death | 1195 (12) | 675 (14) | 330 (16) | 345 (12) | 520 (10) | 233 (11) | 287 (9) |

Presented as number of patients (%).*Subsequent type 1 or type 4b myocardial infarction. †Defined as urgent or emergency percutaneous coronary intervention or coronary artery bypass grafting from discharge to 1 year later. ‡Bleeding Academic Research Consortium type 3 or type 5. §Excludes type 1 or type 4b myocardial infarction.

**Online Table 11: Cox proportional hazards model, coefficients and standard errors for phase and sex interaction**

|  | **Estimate** | **Standard Error** | **z value** | **Pr (>\|z\|)** |
| --- | --- | --- | --- | --- |
| Study phase | 0.045 | 0.08 | 0.563 | 0.573 |
| Sex | 0.125 | 0.075 | 1.657 | 0.098 |
| Age | 0.032 | 0.002 | 15.599 | <0.001 |
| Season: summer | -0.075 | 0.071 | -1.052 | 0.293 |
| Season: autumn | -0.138 | 0.071 | -1.926 | 0.054 |
| Season: winter | -0.109 | 0.072 | -1.519 | 0.129 |
| Days since start of study | -0.002 | 0.001 | -1.835 | 0.067 |
| Deprivation quintile | 0.000 | 0.000 | -3.721 | <0.001 |
| Previous diabetes | -0.261 | 0.063 | -4.15 | <0.001 |
| Previous ischemic heart disease | -0.388 | 0.052 | -7.425 | <0.001 |
| Previous cerebrovascular disease | -0.291 | 0.068 | -4.281 | <0.001 |
| Creatinine | 0.001 | 0.000 | 7.609 | <0.001 |
| Log troponin | 0.114 | 0.011 | 10.758 | <0.001 |
| Study phase and sex interaction | -0.160 | 0.098 | -1.641 | 0.101 |

**Online Table 12: Primary and secondary outcomes after 1 year in patients with type 1 myocardial infarction, stratified by gender and study phase**

|  | Overall | Women | | | Men | | |
| --- | --- | --- | --- | --- | --- | --- | --- |
|  |  | **Overall** | **Validation** | **Implementation** | **Overall** | **Validation** | **Implementation** |
| Number of participants | 5028 | 2010 | 748 | 1262 | 3018 | 1059 | 1959 |
| Primary outcome |  |  |  |  |  |  |  |
| Myocardial infarction* or death from cardiovascular causes | 870 (17) | 375 (19) | 141 (19) | 234 (19) | 495 (16) | 194 (18) | 301 (15) |
| Secondary outcomes |  |  |  |  |  |  |  |
| Myocardial infarction* | 471 (9) | 223 (11) | 93 (12) | 130 (10) | 248 (8) | 101 (10) | 147 (8) |
| Unplanned revascularisation† | 341 (7) | 123 (6) | 47 (6) | 76 (6) | 218 (7) | 93 (9) | 125 (6) |
| All-cause death | 723 (14) | 304 (15) | 115 (15) | 189 (15) | 419 (14) | 169 (16) | 250 (13) |
| Death from cardiovascular causes | 481 (10) | 189 (9) | 64 (9) | 125 (10) | 292 (10) | 112 (11) | 180 (9) |
| Death from cardiac causes | 433 (9) | 165 (8) | 55 (7) | 110 (9) | 268 (9) | 100 (9) | 168 (9) |
| Hospital admission for heart failure | 384 (8) | 174 (9) | 75 (10) | 99 (8) | 210 (7) | 91 (9) | 119 (6) |
| Ischemic stroke | 76 (2) | 37 (2) | 16 (2) | 21 (2) | 39 (1) | 17 (2) | 22 (1) |
| Safety endpoints |  |  |  |  |  |  |  |
| Major hemorrhage‡ | 39 (1) | 15 (1) | 7 (1) | 8 (1) | 24 (1) | 9 (1) | 15 (1) |
| Unplanned hospital admission at 30 days§ | 1626 (32) | 620 (31) | 252 (34) | 368 (29) | 1006 (33) | 450 (42) | 556 (28) |
| Non-cardiovascular death | 242 (5) | 115 (6) | 51 (7) | 64 (5) | 127 (4) | 57 (5) | 70 (4) |

Data are number of patients (%).*Subsequent type 1 or type 4b myocardial infarction. †Defined as urgent or emergency percutaneous coronary intervention or coronary artery bypass grafting from discharge to 1 year later. ‡Bleeding Academic Research Consortium type 3 or type 5. §Excludes type 1 or type 4b myocardial infarction.

**Online Table 13: Primary and secondary outcomes after 1 year in participants, stratified by study phase and gender in patients reclassified by high-sensitivity cardiac troponin I assay**

|  | Overall | Women | | | Men | | |
| --- | --- | --- | --- | --- | --- | --- | --- |
|  |  | **Overall** | **Validation** | **Implementation** | **Overall** | **Validation** | **Implementation** |
| Number of participants | 1771 | 1470 | 612 | 858 | 301 | 108 | 193 |
| Primary outcome |  |  |  |  |  |  |  |
| Myocardial infarction* or death from cardiovascular causes | 236 (13) | 195 (13) | 89 (15) | 106 (12) | 41 (14) | 16 (15) | 25 (13) |
| Secondary outcomes |  |  |  |  |  |  |  |
| Myocardial infarction* | 118 (7) | 90 (6) | 45 (7) | 45 (5) | 28 (9) | 11 (10) | 17 (9) |
| Unplanned revascularisation† | 43 (2) | 26 (2) | 9 (1) | 17 (2) | 17 (6) | 9 (8) | 8 (4) |
| All-cause death | 354 (20) | 311 (21) | 151 (25) | 160 (19) | 43 (14) | 16 (15) | 27 (14) |
| Death from cardiovascular causes | 129 (7) | 112 (8) | 48 (8) | 64 (7) | 17 (6) | 6 (6) | 11 (6) |
| Death from cardiac causes | 91 (5) | 77 (5) | 29 (5) | 48 (6) | 14 (5) | <5 | 11 (6) |
| Hospital admission for heart failure | 204 (12) | 165 (11) | 71 (12) | 94 (11) | 39 (13) | 20 (19) | 19 (10) |
| Ischemic stroke | 41 (2) | 34 (2) | 19 (3) | 15 (2) | 7 (2) | 5 (5) | <5 |
| Safety endpoints |  |  |  |  |  |  |  |
| Major hemorrhage‡ | 16 (1) | 12 (1) | 5 (1) | 7 (1) | <5 | <5 | <5 |
| Unplanned hospital admission at 30 days§ | 453 (26) | 385 (26) | 180 (29) | 205 (24) | 68 (23) | 28 (26) | 40 (21) |
| Non-cardiovascular death | 224 (13) | 198 (13) | 103 (17) | 95 (11) | 26 (9) | 10 (9) | 16 (8) |

Data are number of patients (%).*Subsequent type 1 or type 4b myocardial infarction. †Defined as urgent or emergency percutaneous coronary intervention or coronary artery bypass grafting from discharge to 1 year later. ‡Bleeding Academic Research Consortium type 3 or type 5. §Excludes type 1 or type 4b myocardial infarction.

**Online Table 14. Characteristics of women reclassified by the high-sensitivity cardiac troponin I assay stratified by the uniform threshold.**

|  | No myocardial injury | Reclassified by hs-cTnI | |
| --- | --- | --- | --- |
|  | **≤16 ng/L** | **16 to 26 ng/L** | **>26 to contemporary threshold** |
| Number of participants | 17571 | 901 | 569 |
| Age, years | 59.8 (17.1) | 76.3 (12.9) | 76.3 (13.9) |
| Presenting complaint* |  |  |  |
| Chest pain | 12896 (83.9) | 525 (66.7) | 344 (65.5) |
| Dyspnoea | 507 (3.3) | 90 (11.4) | 71 (13.5) |
| Palpitation | 550 (3.6) | 37 (4.7) | 27 (5.1) |
| Syncope | 754 (4.9) | 68 (8.6) | 35 (6.7) |
| Other | 670 (4.4) | 67 (8.5) | 48 (9.1) |
| Previous medical conditions |  |  |  |
| Myocardial infarction | 995 (5.7) | 91 (10.1) | 73 (12.8) |
| Ischemic heart disease | 3429 (19.5) | 316 (35.1) | 210 (36.9) |
| Cerebrovascular disease | 857 (4.9) | 117 (13.0) | 61 (10.7) |
| Diabetes mellitus | 785 (4.5) | 97 (10.8) | 66 (11.6) |
| Previous revascularisation |  |  |  |
| PCI | 835 (4.8) | 64 (7.1) | 50 (8.8) |
| CABG | 124 (0.7) | 13 (1.4) | 11 (1.9) |
| Medications at presentation |  |  |  |
| Aspirin | 3940 (22.4) | 336 (37.3) | 219 (38.5) |
| P2Y12 inhibitor | 1354 (7.7) | 146 (16.2) | 69 (12.1) |
| Dual anti-platelet therapy† | 382 (2.2) | 35 (3.9) | 26 (4.6) |
| Statin | 6051 (34.4) | 481 (53.4) | 310 (54.5) |
| ACE or ARB | 4845 (27.6) | 368 (40.8) | 246 (43.2) |
| Beta-blocker | 4272 (24.3) | 342 (38.0) | 207 (36.4) |
| Oral anticoagulant‡ | 962 (5.5) | 129 (14.3) | 74 (13.0) |
| Loop diuretic | 2015 (11.5) | 318 (35.3) | 188 (33.0) |
| Proton pump inhibitor | 7733 (44.0) | 458 (50.8) | 284 (49.9) |
| Calcium channel blocker | 2263 (12.9) | 159 (17.6) | 104 (18.3) |
| Nicorandil | 681 (3.9) | 51 (5.7) | 39 (6.9) |
| Ivabradine | 118 (0.7) | 14 (1.6) | 12 (2.1) |
| Spironolactone | 250 (1.4) | 44 (4.9) | 23 (4.0) |
| Electrocardiogram result§ |  |  |  |
| Normal | 13 (52.0) | 293 (44.2) | 205 (45.1) |
| Physiological parameters§ |  |  |  |
| Heart rate, beats per minute | 74.4 (23.3) | 86.4 (26.4) | 87.1 (27.9) |
| Systolic blood pressure, mmHg | 135.9 (24.3) | 144.6 (28.5) | 142.3 (26.8) |
| GRACE risk score | 129.0 (31.0) | 139.6 (29.1) | 146.7 (34.4) |
| Hematology and clinical chemistry measurements |  |  |  |
| Haemoglobin, g/L | 130.4 (18.1) | 125.5 (20.7) | 123.6 (23.2) |
| eGFR, ml/min | 55.7 (10.5) | 47.5 (14.7) | 47.0 (14.8) |
| Peak hs-cTnI, ng/L | 2.0 (1.0 - 4.8) | 20.5 (18.1 - 23.0) | 33.0 (29.0 - 40.8) |
| Serial high-sensitivity cardiac troponin I, % ¶ | 7275 (41.4) | 494 (54.8) | 343 (60.3) |
| Adjudicated diagnosis# |  |  |  |
| Type 1 MI | 0 (0.0) | 219 (28.3) | 182 (36.6) |
| Type 2 MI | 0 (0.0) | 115 (14.9) | 80 (16.1) |
| Non-ischemic myocardial injury | 0 (0.0) | 438 (56.7) | 235 (47.3) |

*Presented as number of patients (%), mean (SD) or median (IQR). Abbreviations: ACE = angiotensin converting enzyme; ARB = angiotensin receptor blocker; eGFR = estimated glomerular filtration rate; PCI = percutaneous coronary intervention; CABG = coronary artery bypass grafting; UDMI = Universal Definition of Myocardial Infarction.*

**A presenting symptom was missing in 5615 (12%) patients. †Two medications from aspirin, clopidogrel, prasugrel or ticagrelor. ‡Includes warfarin or novel oral anti-coagulants. §Electrocardiographic data was available in 1377 (78%) of reclassified patients and 6470 (75%) of identified patients. ¶Defined as two or more tests within 24 hours from presentation. #The adjudication panel were able to achieve a consensus diagnosis in 9,115 (88%) patients with hs-cTnI concentrations above the sex-specific 99th centile.*

**Online Table 15. Management of women reclassified by the high-sensitivity cardiac troponin I assay stratified by the uniform threshold.**

|  | No myocardial injury | Reclassified by hs-cTnI | |
| --- | --- | --- | --- |
|  | **≤16 ng/L** | **16 to 26 ng/L** | **>26 to contemporary threshold** |
| Number of participants | 17571 | 901 | 569 |
| Duration of hospital stay, hrs | 5.1 (3.2 - 21.6) | 29.9 (6.3 - 112.9) | 44.5 (12.1 - 138.3) |
| Coronary angiography | 181 (1.0) | 50 (5.5) | 50 (8.8) |
| PCI or CABG | 75 (0.4) | 29 (3.2) | 24 (4.2) |
| New anti-platelet drug | 759 (4.3) | 108 (12.0) | 94 (16.5) |
| New DAPT | 190 (1.1) | 60 (6.7) | 61 (10.7) |
| New statin therapy | 453 (2.6) | 43 (4.8) | 39 (6.9) |
| New ACE or ARB | 310 (1.8) | 48 (5.3) | 36 (6.3) |
| New beta-blocker | 879 (5.0) | 118 (13.1) | 66 (11.6) |
| New oral anticoagulant | 360 (2.0) | 63 (7.0) | 36 (6.3) |

*Presented as number of patients (%) or median (IQR). Abbreviations: ACE = angiotensin converting enzyme; ARB = angiotensin receptor blocker; DAPT= dual anti-platelet therapy; PCI = percutaneous coronary intervention; CABG = coronary artery bypass grafting.*

**Comparison between validation and implementation phase. †P value <0.05 comparing women and men.*

**Online Table 16. Outcomes of women reclassified by the high-sensitivity cardiac troponin I assay stratified by the uniform threshold.**

|  | No myocardial injury | Reclassified by hs-cTnI | |
| --- | --- | --- | --- |
|  | **≤16 ng/L** | **16 to 26 ng/L** | **>26 to contemporary threshold** |
| Number of participants | 17571 | 901 | 569 |
| Primary outcome |  |  |  |
| Myocardial infarction* or death from cardiovascular causes | 333 (1.9) | 121 (13.4) | 74 (13.0) |
| Secondary outcomes |  |  |  |
| Myocardial infarction* | 134 (0.8) | 56 (6.2) | 34 (6.0) |
| Unplanned revascularisation† | 80 (0.5) | 11 (1.2) | 15 (2.6) |
| All-cause death | 813 (4.6) | 191 (21.2) | 120 (21.1) |
| Death from cardiovascular causes | 208 (1.2) | 69 (7.7) | 43 (7.6) |
| Death from cardiac causes | 111 (0.6) | 53 (5.9) | 24 (4.2) |
| Hospital admission for heart failure | 266 (1.5) | 102 (11.3) | 63 (11.1) |
| Ischemic stroke | 146 (0.8) | 24 (2.7) | 10 (1.8) |
| Safety endpoints |  |  |  |
| Major hemorrhage‡ | 31 (0.2) | <10 | <10 |
| Unplanned hospital admission at 30 days§ | 2542 (14.5) | 244 (27.1) | 141 (24.8) |
| Non-cardiovascular death | 605 (3.4) | 122 (13.5) | 76 (13.4) |

*Presented as number of patients (%).*Subsequent type 1 or type 4b myocardial infarction. †Defined as urgent or emergency percutaneous coronary intervention or coronary artery bypass grafting from discharge to 1 year later. ‡Bleeding Academic Research Consortium type 3 or type 5. §Excludes type 1 or type 4b myocardial infarction.*

**Figure Legends**

**Online Figure 1. CONSORT Diagram of the Trial and Populations**

Between June 10, 2013, and March 3, 2016, 48 282 consecutive patients with suspected acute coronary syndrome met the trial inclusion criteria and were included in the analysis of the primary outcome. The trial concluded on March 3, 2017, after a minimum follow-up period of 1 year.

**Online Figure 2. Management of patients above and below the median age of all patients with myocardial injury (73 years) during the index hospitalisation and odds ratio of myocardial infarction or cardiovascular death at 1 year stratified by treatment received and by sex**

Odds ratio was adjusted for hospital site (fitted as a random effect), season, time of presentation from the start date of the trial, age, sex and study phase as an interaction term, previous history of diabetes mellitus, ischemic heart disease or cerebrovascular disease, hs-cTnI, creatinine concentrations and social deprivation.

**Online Figure 3. Management of patients with type 1 myocardial infarction during the index hospitalisation and odds ratio of myocardial infarction or cardiovascular death at 1 year stratified by treatment received and by sex**

**(Top)** Odds ratio of myocardial infarction and cardiovascular death in patients receiving each treatment modality compared to those who did not in all patients and stratified by sex. Odds ratio was adjusted for hospital site (fitted as a random effect), season, time of presentation from the start date of the trial, age, sex and study phase as an interaction term, previous history of diabetes mellitus, ischemic heart disease or cerebrovascular disease, hs-cTnI, creatinine concentrations and social deprivation. **(Middle)** Treatment commenced during index hospitalisation in all patients and stratified by sex. **(Bottom)** Pre-existing medications and treatment prior to index presentation in all patients and stratified by sex.

**Online Figure 1.**

**Online Figure 2.**

**Online Figure 3.**

**References**

1. Mehran R, Rao SV, Bhatt DL, et al. Standardized bleeding definitions for cardiovascular clinical trials: a consensus report from the Bleeding Academic Research Consortium. Consensus Development Conference presented at Circulation; Jun 14, 2011.

2. Denaxas SC, George J, Herrett E, et al. Data resource profile: cardiovascular disease research using linked bespoke studies and electronic health records (CALIBER). *Int J Epidemiol.* 2012;41(6):1625-1638.

3. The Scottish Index of Multiple Deprivation. Scottish Government. Accessed August 1st 2018, [www.gov.scot/Topics/Statistics/SIMD](file:///C:\Users\nickmills\Desktop\JACC%20rebuttal\Final\Penultemate\www.gov.scot\Topics\Statistics\SIMD).

4. Granger CB, Goldberg RJ, Dabbous O, et al. Predictors of hospital mortality in the global registry of acute coronary events. *Arch Intern Med.* 2003;163(19):2345-2353.

5. Mills NL, Churchhouse AMD, Lee KK, et al. Implementation of a sensitive troponin I assay and risk of recurrent myocardial infarction and death in patients with suspected acute coronary syndrome. *JAMA.* 2011;305(12):1210-1216.

6. Shah ASV, Griffiths M, Lee KK, et al. High sensitivity cardiac troponin and the under-diagnosis of myocardial infarction in women: prospective cohort study. *BMJ.* 2015;350:g7873.

7. Shah ASV, Anand A, Strachan FE, et al. High-sensitivity troponin in the evaluation of patients with suspected acute coronary syndrome: a stepped-wedge, cluster-randomised controlled trial. *The Lancet.* 2018;392(10151):919-928.

8. von Elm E, Altman DG, Egger M, Pocock SJ, Gøtzsche PC, Vandenbroucke JP. The Strengthening the Reporting of Observational Studies in Epidemiology (STROBE) statement: guidelines for reporting observational studies. *The Lancet.* 2007;370(9596):1453-1457.
